# A novel role of the organizer gene *Goosecoid* as an inhibitor of Wnt/PCP-mediated convergent extension in *Xenopus* and mouse

**DOI:** 10.1038/srep43010

**Published:** 2017-02-21

**Authors:** Bärbel Ulmer, Melanie Tingler, Sabrina Kurz, Markus Maerker, Philipp Andre, Dina Mönch, Marina Campione, Kirsten Deißler, Mark Lewandoski, Thomas Thumberger, Axel Schweickert, Abraham Fainsod, Herbert Steinbeißer, Martin Blum

**Affiliations:** 1 University of Hohenheim, Garbenstr. 30, 70599 Stuttgart, Germany; 2 Genetics of Vertebrate Development Section, Cancer and Developmental Biology Lab, National Cancer Institute, National Institutes of Health, Frederick, MD 21702, USA; 3 Department of Developmental Biology and Cancer Research, Institute for Medical Research Israel-Canada, Hebrew University, Jerusalem 9112102, Israel; 4 Institute of Human Genetics, University Hospital Heidelberg, Im Neuenheimer Feld 366, 69120 Heidelberg, Germany

## Abstract

*Goosecoid (Gsc*) expression marks the primary embryonic organizer in vertebrates and beyond. While functions have been assigned during later embryogenesis, the role of *Gsc* in the organizer has remained enigmatic. Using conditional gain-of-function approaches in *Xenopus* and mouse to maintain *Gsc* expression in the organizer and along the axial midline, neural tube closure defects (NTDs) arose and dorsal extension was compromised. Both phenotypes represent convergent extension (CE) defects, arising from impaired Wnt/planar cell polarity (PCP) signaling. Dvl2 recruitment to the cell membrane was inhibited by Gsc in *Xenopus* animal cap assays and key Wnt/PCP factors (*RhoA, Vangl2, Prickle, Wnt11*) rescued Gsc-mediated NTDs. Re-evaluation of endogenous *Gsc* functions in MO-mediated gene knockdown frog and knockout mouse embryos unearthed PCP/CE-related phenotypes as well, including cartilage defects in *Xenopus* and misalignment of inner ear hair cells in mouse. Our results assign a novel function to *Gsc* as an inhibitor of Wnt/PCP-mediated CE. We propose that in the organizer *Gsc* represses CE as well: *Gsc*-expressing prechordal cells, which leave the organizer first, migrate and do not undergo CE like the *Gsc*-negative notochordal cells, which subsequently emerge from the organizer. In this model, *Gsc* provides a switch between cell migration and CE, i.e. cell intercalation.

During development, invertebrate and vertebrate embryos alike elongate and narrow their anterior-posterior (AP) axis by convergent extension (CE). CE is driven by intercalation of bipolar cells perpendicular to the previously established AP axis, necessitating a perfect coordination between spatial cues and cellular behavior. In *Drosophila* it has been shown that positional AP information, encoded by *Eve, Runt* and localized *Toll*-receptor expression, is directly translated into germ band CE^1^. Likewise, AP-patterning was shown to be directly linked to CE movements in explanted chordamesoderm of *Xenopus* embryos^2^. Molecular cues, which control and orient CE relative to the AP axis, have not been described in vertebrate embryos. How the spatial patterning is maintained and reinforced in the highly dynamic environment of the elongating and developing vertebrate embryo has yet to be defined.

The vertebrate body plan is established during gastrulation through the activity of the primary embryonic organizer (Spemann organizer), a specialized group of cells located at the amphibian dorsal lip of the blastopore or homologous structures in other vertebrates (node in birds and mammals, embryonic shield in fish^3^). Organizer transplantation to the opposite, ventral side of the gastrula embryo induces the formation of a secondary axis, in which neighboring ventral cells adopt both a dorsal fate and undergo gastrulation movements^4^. Expression of the homeobox transcription factor gene *Goosecoid (Gsc*) marks Spemann’s organizer in vertebrates and beyond^5^,^6^. Upon ectopic expression on the ventral side, i.e. opposite to its normal site of action, *Gsc* efficiently induces the formation of secondary embryonic axes in *Xenopus*^7^. This remarkable ability to mimic Spemann’s organizer in gain-of-function experiments is readily explained by its well characterized ability to transcriptionally repress target genes identified in mouse, frog and zebrafish, including *Wnt8a* and *BMP4* pathway components^8^,^9^,^10^,^11^,^12^,^13^,^14^,^15^,^16^,^17^,^18^. In stark contrast, *Gsc* knockout mouse embryos lack gastrulation defects^19^,^20^, as do frog and fish embryos with impaired *Gsc* function^15^,^16^,^21^,^22^. This lack of a gastrulation phenotype is likely explained by functional redundancy with other factors expressed in the organizer, which await identification.

Yet there may be additional *Gsc* functions in the organizer. A number of studies suggested a general role of *Gsc* in cell migration during development and disease that is not explained by its role as a transcriptional repressor of BMP4 and Wnt8 targets. Lineage labeling and video microscopy of *Gsc*-injected embryos revealed enhanced anterior migration of posterior cells^23^. *Gsc* was also able to enhance the migratory behavior of cultured embryonic frog head mesenchymal cells^24^. In tumor cells, *Gsc* expression correlated with enhanced migratory activity as well^25^. Together these data point to a possible role of Gsc in mediating cellular behavior.

The early embryonic expression pattern of *Gsc* in vertebrate embryos is in agreement with such a function. The initial transcription in the organizer tissue itself is very transient. As axial mesodermal cells (prechordal plate and notochord) begin to leave the organizer in rostral direction, *Gsc* expression remains active in prechordal cells but ceases in the resident organizer tissue and the notochord^10^,^26^,^27^. Segregation of organizer-derived cells into these two cell populations is accompanied by differences in cell behavior and gene expression: *Gsc* marks the prechordal cells, characterized by single cell migration, while *Brachyury* is expressed and instrumental for CE in the notochord^28^,^29^,^30^,^31^.

Based on this dichotomy we hypothesize that *Gsc* plays a role in prechordal cells to promote migration and to inhibit CE. In order to test this hypothesis, we performed conditional gain-of-function experiments in mouse and *Xenopus*. Our experiments resulted in CE-phenotypes in both species, including neural tube closure and axial elongation defects. Rescue of *Gsc*-induced CE phenotypes by co-expression of planar cell polarity (PCP) pathway components suggested a novel function of *Gsc* as a negative regulator of PCP-mediated CE. Loss-of function experiments showed that *Gsc* impaired bipolar elongation of cells in Meckel’s cartilage in *Xenopus* and affected the alignment of hair cells in the inner ear of *Gsc* knockout mouse embryos. Based on these results we propose a novel role of Gsc as inhibitor of PCP-mediated CE.

## Results

### Sustained *Gsc* expression along the axial midline interferes with CE and causes neural tube and blastopore closure defects in *Xenopus*

*Gsc* expression in the organizer ceases with the exit of the first cell population, which migrates anteriorly and constitutes the prechordal mesoderm. Our hypothesis predicts that a sustained activity of Gsc along the subsequently emerging notochord interferes with the cellular behavior of these cells, namely CE. In order to ectopically express Gsc in a tightly controlled temporal and spatial manner, we employed a previously described inducible Gsc protein^32^. In short, a construct was used, in which the Gsc coding sequence was fused to the ligand binding domain of the glucocorticoid receptor (GR). In the absence of the synthetic ligand dexamethasone (dex), Gsc-GR localizes to the cytoplasm and remaines inactive, while ligand addition results in a conformational change, nuclear entry and onset of Gsc function as a transcriptional repressor^32^. Functionality of the construct was demonstrated by dex treatment of ventrally injected specimens, which led to double axis induction in 14/24 cases, i.e. at frequencies described previously^32^ (not shown).

**Figure 1:**
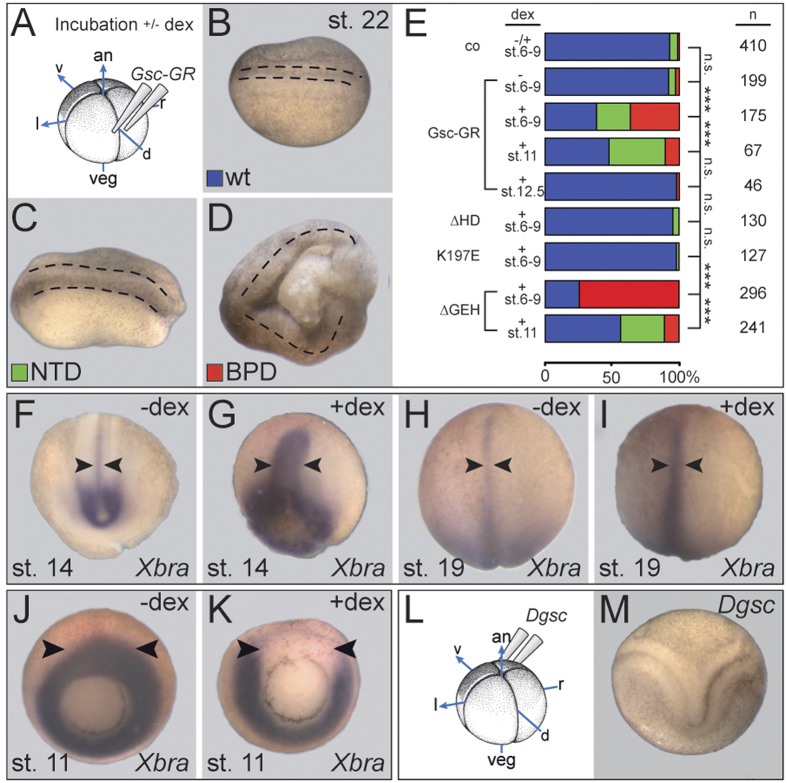
Gsc-mediated CE phenotypes in *Xenopus*. (**A**) Experimental design. Specimens were injected with *Gsc-GR* into the dorsal marginal region of the 4-cell embryo and cultured to the stages indicated, with or without addition of dex. (**B–E**) *Gsc-GR* induced NTD and BPD in whole embryos. Specimens were scored for wt appearance (blue; **B**), NTD (green; **C**) and BPD (red; **D**). Anterior is to the left in (**B**–**D**). (**E**) Compilation of results. Note that *Gsc-GR* caused CE phenotypes in a highly significant proportion of embryos, but only when activated before and during gastrulation. Note also that deletion of the homeodomain (∆HD) or altering the DNA-binding specificity (K197E) prevented BPD/NTD-induction, while the repression domain GEH was not required for BPD/NTD. (**F–I**) Impaired CE of the notochord upon sustained dorsal *Gsc-GR* expression. Note that the notochord was wider and shorter in dex-treated (**G,I**) as opposed to untreated (**F**,**H**) specimens, both at stage 14 (**F,G**) and stage 19 (**H,I**). (**J,K**) Repression of *Xbra* transcription on the dorsal side upon *Gsc-GR* activation. (**L,M**) Double axis formation (**M**) following ventral injections of *Dgsc* mRNA into 4-cell *Xenopus* embryos (**L**).

Targeting of Gsc-GR to the dorsal midline was achieved by microinjection of synthetic mRNA into the marginal region of the two dorsal blastomeres of the 4-cell embryo (Fig. 1A). Analysis of a co-injected lineage tracer confirmed delivery to the notochord and floor plate, which cannot be targeted separately in such experiments (not shown). No phenotypic changes were observed in the absence of dex (Fig. 1B,E), while ligand addition between cleavage and blastula stages (st. 6–9) resulted in a high percentage of embryos with neural tube closure defects (NTDs; Fig. 1C,E; Table S1). More severe blastopore closure defects (BPD^33^) were observed as well (Fig. 1D,E; Table S1). In these cases, the dorsal midline was disrupted, which resulted in cup-shaped morphologies (Fig. 1D). The overall percentage of affected embryos dropped when dex was added during gastrulation, and very few malformations were recorded when Gsc-GR was activated during late gastrula/early neurula stages (Fig. 1E; Table S1 and data not shown). Development of BPD and NTD depended on the presence of the homeodomain (HD) as well as the paired-type DNA binding specificity of Gsc (lysine in position 50 of the HD), while the repression domain (eh1/GEH) was not required for NTD/BPD induction (Fig. 1E). A slight but non-significant delay in neural tube closure was observed in a proportion of specimens (not shown). Sustained *Gsc* expression along the dorsal midline thus interfered with blastopore and neural tube closure, processes known to depend on CE^34^,^35^.

*Xbra* mRNA transcription serves as a readout of CE in the notochord, which narrows and lengthens concomitantly with neural tube closure^36^. In order to assess whether notochordal CE was affected by sustained *Gsc* expression as well, we analyzed *Xbra* in less severely affected dex-treated specimens without BPD. In the absence of dex, the notochord was elongated and narrow during neurula stages. Activation of ectopic Gsc activity, however, resulted in shortened and widened *Xbra* expression domains (Fig. 1F–I), in agreement with CE defects in the notochord. While the expression level of *Xbra* in the notochord was not affected, we expected a repression of *Xbra* transcription by Gsc during gastrulation, in line with the reported role of Gsc as a repressor of *Brachyury* in the prechordal mesoderm^10^,^11^,^13^. Analysis at late gastrula (stage 11) demonstrated that repression of *Xbra* in dex-treated specimens took place but was restricted to the injection site (Fig. 1K; 35/74, 47.3%). In the absence of dex, *Gsc-GR* injected embryos showed wildtype (wt) *Xbra* expression around the blastopore (arrowheads, Fig. 1J; 48/51, 94.1%).

**Figure 2:**
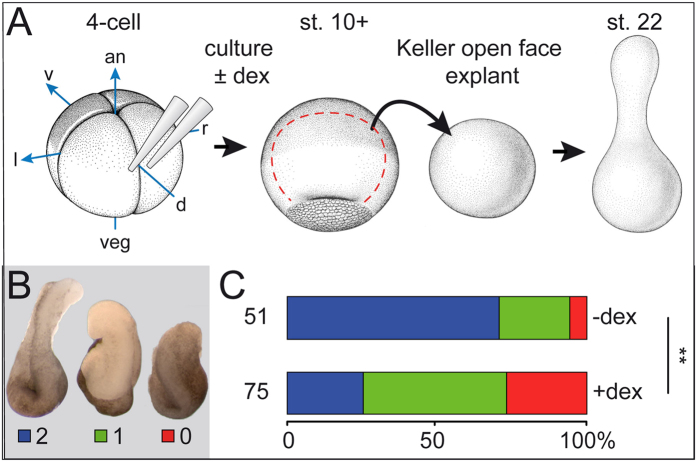
Gsc inhibits CE in Keller open face explants. (**A–C**) CE defects in Keller open face explants (schematically depicted in (**A**) upon activation of *Gsc-GR*. (**B**) Explants were classified as class 2 (blue) when extensions showed a constriction (left), as class 1 (green) when elongation occurred without constriction (middle), and as class 0 (red) when no elongation ensued (right)^38^. an, animal; uninj., uninjected control; d, dorsal; l, left; r, right; v, ventral; veg, vegetal. (**C**) Summary of results.

In order to assess the effects of Gsc on CE in a semi-quantitative manner, we turned to Keller open-face explants, which have been used in the past to investigate notochord CE in *ex vivo* assays^37^ (Fig. 2A). Dorsal marginal zone tissue was isolated at stage 10–10.5 from *Gsc-GR*-injected embryos, which were incubated in the presence or absence of dex from stage 6/7 onwards, and scored for CE when un-injected siblings reached stage 22 (Fig. 2A–C). CE was classified into three categories^38^, with class 0 representing explants without elongation, class 1 containing elongated specimens, and class 2 explants which in addition displayed a constriction (Fig. 2B). In the absence of dex, more than 90% of explants elongated, with the majority of specimens falling into class 2 (36/51; 70.6%). In contrast, CE in dex-treated explants was severely compromised, with significantly reduced class 2 extensions (19/75), the relative majority of specimens elongating without constriction and about 25% not elongating at all (class 1; 36/75, 48%; Fig. 2C).

In order to investigate if and how sustained Gsc expression along the dorsal midline interfered with cell fate determination, i.e. with neural induction and mesodermal patterning, mRNA transcription of neural (*Ncam*) and somitic (*MyoD*) marker genes was analyzed. Both genes were expressed in specimens displaying BPDs upon dex treatment, even though somites did not epithelialize into the typical chevron-shaped patterns of control specimens (Fig. S1A–D). Sustained expression of Gsc on the dorsal side of *Xenopus* embryos thus did not interfere with specification of neural and mesodermal tissue, but inhibited CE in the notochord.

**Figure 3:**
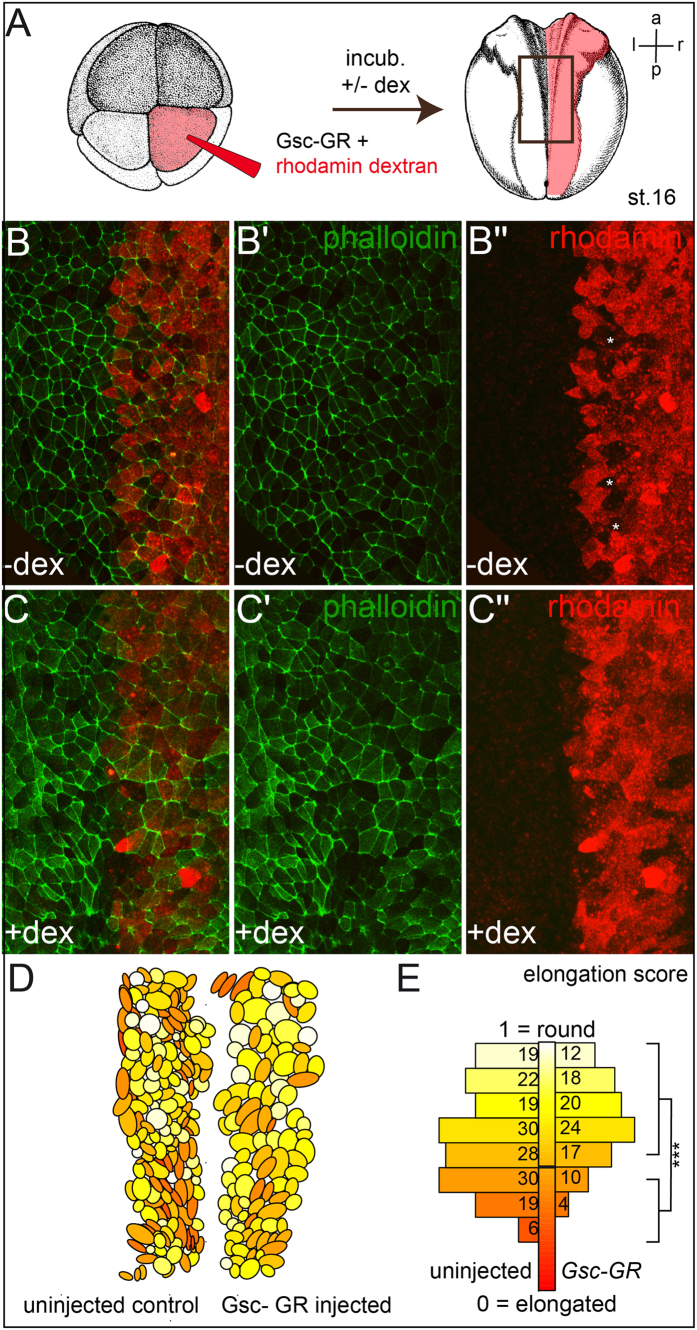
Gsc compromises bipolar elongation of neural plate cells. (**A**) Targeted injection scheme of *Gsc-GR* and linage tracer (rhodamine red) into the right side of the neural plate (**B,C**). Drawings taken from Xenbase (www.xenbase.org/anatomy/alldevo.do)^97^. (**D,E**) Analysis of cell elongation. The color gradient ranging from pale yellow (round, width = length, 1) to dark red (elongated, 0) exemplifies the change from bipolar cells on the un-injected (right) side towards rounded cells upon activation of Gsc-GR (**D**). (**E**) Significant decrease of percentage of elongated cells (elongation score <1/2) after *Gsc-GR* missexpression. a, anterior; l, left; p, posterior; r, right.

To analyze whether NTDs were caused by impaired CE as well, we investigated a potential role of Gsc in cell shape changes in the neuroectoderm. A prerequisite of CE is that cells polarize, i.e. elongate and adopt a bipolar morphology. *Gsc-GR* was targeted to the neuroectoderm by microinjecting synthetic mRNA to the A1 lineage of 8-cell embryos. Rhodamine dextran was co-injected as a linage tracer, and injections were performed unilaterally in order to provide for an internal control on the un-injected contralateral side (Fig. 3A). Injected specimens were incubated until mid-neurula stages (stage 16), fixed and processed for cell shape assessment via phalloidin-staining of the actin cytoskeleton. In the absence of dex, cell morphologies appeared indistinguishable on both sides, while Gsc activation resulted in less elongated, rounder cells (Fig. 3B–D). To quantitate this effect, the length-to-width ratio was determined and expressed as elongation score, with a value of 1 representing a round cell and 0 a hypothetical elongated cell without width. The results from a representative specimen are depicted in Fig. 3E. On the Gsc-GR side a significant decrease of cells displaying a score of <0.5 was observed (14/105 or 13% on the Gsc-GR injected side, and 55/173 or 32% on the control side). In addition, unlabeled cells in between the injected rhodamine dextran-positive cells, which likely represent intercalation events, were observed on un-injected and untreated control sides (asterisks in Fig. 3B). Upon Gsc activation, no such unlabeled cells were found (Fig. 3C). In some explants, cell numbers were slightly (and non-significantly) reduced (not shown), however, cell proliferation and apoptosis were not affected by Gsc-GR activation (Fig. S2). The occasionally observed alterations of cell numbers may be caused by dex treatment, as previously reported^39^. These results strongly suggest that NTDs in frog tadpoles were due to impaired CE as well, caused by a lack of bipolar cell polarization in Gsc-misexpressing neuroectodermal cells.

Finally, we wondered whether this novel function of Gsc as an inhibitor of CE was evolutionary conserved. *Gsc* represents an ancient member of the metazoan toolkit of animal embryogenesis which is present from radiata (cnidarians; hydra^6^,^40^) to lophotrochozoans^41^, ecdysozoans (e.g. Drosophila) and deuterostomians alike. In all cases, the homeodomain and the N-terminal repression domain are highly conserved^42^,^43^. We chose to analyze Drosophila *Gsc*, which was previously shown to be able to rescue the dorsal axis of UV-treated ventralized *Xenopus* embryos^44^. In line with these experiments, *Dgsc* was able to induce double axis formation upon ventral injection (Fig. 1L, M; 24/25, 96%). Dorsal injections of *Dgsc*, however, had no effect on neural tube or blastopore closure (100/100, not shown), indicating that the novel function of Gsc described here as a repressor of CE arose later in evolution and may be independent of its function as a transcriptional repressor.

### Expression of *Gsc* in the entire mouse primitive streak results in NTD and compromises axial extension

**Figure 4:**
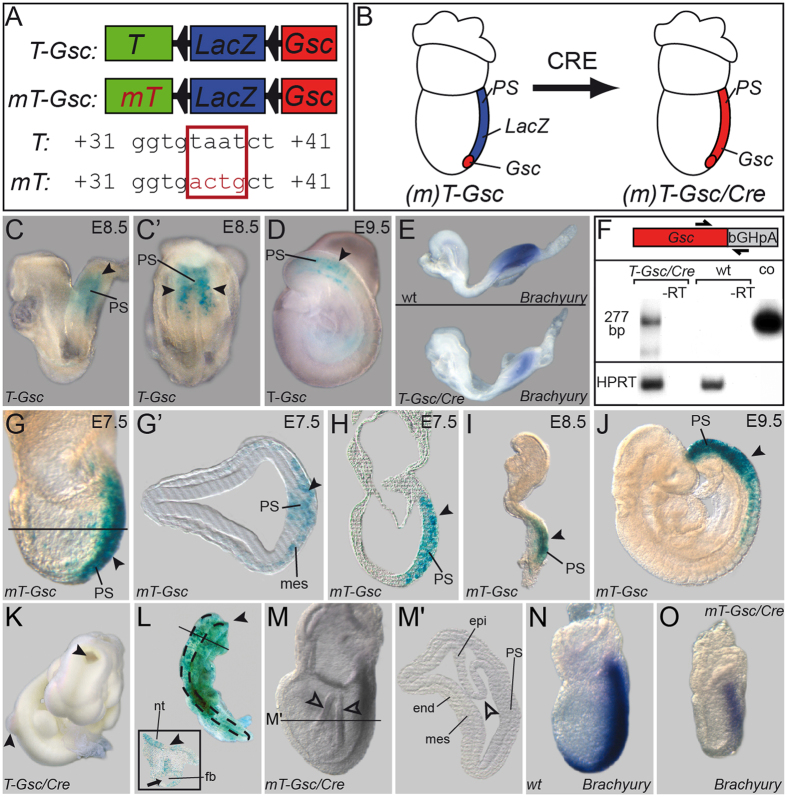
*Gsc*-mediated CE phenotypes in the mouse. Conditional misexpression of *Gsc* in the entire primitive streak of the mouse. (**A**) Constructs used to generate transgenic mouse lines. *T*, wt *Brachyury* streak enhancer; *mT*, mutant enhancer not repressed by Gsc; triangles, loxP sites. (**B**) Schematic depiction of *Gsc* (red) and LacZ (blue) expression at E7.5 before (left) and after (right) Cre-mediated recombination. (**C,D**) LacZ expression (arrowheads) in the primitive streak (PS) mesoderm of E8.5 (lateral view in **C**, posterior view in **C’**) and E9.5 (**D**) *T-Gsc* embryos. (**E**) Reduced *Brachyury* mRNA expression upon transgene activation (*T-Gsc/Cre*, lower panel) compared to wt embryo (upper panel). (**F**) Detection of transgenic *Gsc* mRNA by RT-PCR from *T-Gsc/Cre* and wt E8.5 embryos. A 277 bp fragment specific for transgenic *Gsc* mRNA was amplified using a *Gsc* primer and a primer derived from the bovine growth hormone polyadenylation (bGHpA) signal present in the construct. Note that no signal was detected in wt embryos, and that a band identical in size to one amplified from the *T-Gsc* control plasmid was seen in *T-Gsc/Cre* embryos. (**G–J**) LacZ expression (arrowheads) in the PS mesoderm of E7.5 (**G,H**) plane of histological section G’ indicated in (**G**), E8.5 (**I**) and E9.5 (**J**) *mT-Gsc* embryos. (**K**) Cranial and caudal NTD (arrowheads) in E10.5 *T-Gsc/Cre* embryo. (**L**) Craniorachischisis in chimeric E10.5 embryo generated from ES cells expressing *LacZ* and *Gsc*. Note that, except for the forebrain region (arrow; cross section shown in inset), the entire neural tube stayed open (arrowheads). (**M**) Malformation of *mt-Gsc/Cre* gastrula embryo. Note irregular folding of epiblast (open arrowheads). (**M’**) Histological section at level indicated in (**M**). (**N,O**) Repression of *Brachyury* transcription in *mT-Gsc/Cre* (**O**) compared to wt (**N**) E7.5 embryos. end, endoderm; epi, epiblast; fb, forebrain; mes, mesoderm; nt, neural tube; PS, primitive streak.

Next we wondered whether this novel role of Gsc to repress CE was conserved among the vertebrates. To investigate this possibility, we expressed *Gsc* in the entire primitive streak of mouse embryos using a conditional approach^45^. Construct *T-Gsc* contained the 650 bp primitive streak enhancer of the mouse *Brachyury (T)* gene^46^, followed by a floxed LacZ gene and the mouse *Gsc* coding sequence (Fig. 4A). Construct *mT-Gsc* was identical, except that the Gsc-binding site in the *Brachyury* streak enhancer was mutated to prevent Gsc-mediated transgene repression^11^. Thus, *T-Gsc* should result in moderate transgene expression, creating a scenario resembling the endogenous *Gsc* gene, where Gsc negatively autoregulates its own expression^47^. *mT-Gsc*, in contrast, should allow for pronounced and sustained ectopic *Gsc* expression in the primitive streak mesoderm and descendants thereof. Transgenic *T-Gsc* mouse lines moderately expressed the LacZ reporter gene in the nascent primitive streak mesoderm from E7.5 onwards (Fig. 4C,D and data not shown). Much stronger LacZ staining was found in embryos of *mT-Gsc* lines, as expected (Fig. 4G–J).

To study the phenotypes induced by ectopic Gsc activity, mice were mated to the *deleter* line, which expresses the CRE-recombinase ubiquitously from blastocyst stages onwards^48^ (Fig. 4B). First, the effects of moderate *Gsc* misexpression were assessed. Transgenic *T-Gsc* embryos analyzed from E7.0-E9.0 were morphologically indistinguishable from wt specimens (not shown). *Brachyury* expression in the primitive streak was reduced (Fig. 4E), demonstrating that the transgenic Gsc protein was functional. Transgenic *Gsc* expression was verified by RT-PCR (Fig. 4F). Phenotypic effects, however, were encountered in 44/197 (22.3%) of transgenic embryos analyzed at E9.5-E10.5. Affected specimens in all cases were characterized by cranial NTDs, while 10/44 in addition showed spina bifida (Fig. 4K). In order to prove the specificity of Gsc-induced NTDs, we generated chimeric mouse embryos by blastocyst injection of ES cells stably expressing Gsc and LacZ. Embryos were analyzed at E9.5-E10.5 to assess NTDs. In control chimeric embryos, derived from injection of ES cells expressing only LacZ, no NTDs were observed (not shown). *Gsc/LacZ* chimeras, in contrast, were characterized by a high percentage of NTDs which were encountered in 22/27 specimens (81.5%) generated in five experiments. Of these, two chimeric embryos were characterized by a lack of closure along the entire cranio-caudal axis except for the forebrain region (craniorachischisis; Fig. 4L). Together these data demonstrated that NTDs induced from moderate level overexpression of *Gsc* in the primitive streak of transgenic *T-Gsc/Cre* embryos represented a *Gsc*-specific gain-of-function phenotype.

High level ectopic *Gsc* expression from Cre-mediated activation of *mT-Gsc* resulted in much earlier phenotypes. At E8.5 only very few but severely malformed embryos were recovered (not shown). E7.5 *mT-Gsc/Cre* embryos expressed various levels of *Gsc* transcripts. Compared to wt embryos, *mT-Gsc* specimens generally revealed *Gsc* expression domains that were more intensely stained and extended towards the caudal primitive streak (Fig. S3A–D). E7.5 specimens displayed a range of deficiencies that can roughly be grouped into two categories. A typical example of a mildly affected embryo, which was seen in about 60% of cases, is shown in Fig. 4M. The overall size did not differ significantly from wt, however, the epiblast appeared folded-up, which was more obvious in sections (arrowhead in Fig. 4M’). Primitive streak and mesoderm were clearly discernible. Severely affected embryos, in contrast, were characterized by egg cylinders that appeared hardly elongated at all and were approximately half the size of wt specimens (Fig. S3J,L).

The lack of axial elongation suggested that notochordal cells did not form or did not undergo CE. To investigate these options, E7.5 *mT-Gsc/Cre* embryos were analyzed morphologically, histologically and for marker gene expression. Scanning electron microscopy demonstrated that mutant embryos lacked the ciliated epithelium of the posterior notochord (PNC) at the distal tip of the egg cylinder, that is also known as ventral node^26^ (Fig. S3E,F). The notochordal plate, i.e. the anterior extension of the PNC from which the notochord develops, was consistently absent in severely affected embryos as well (Fig. S3F and data not shown). To analyze axial mesoderm formation, the notochordal marker genes *Brachyury* and *Noto* were studied (Fig. 4N,O; Fig. S3G,H). Both genes were clearly down-regulated. Residual mRNAs were found in the primitive streak (*Brachyury*; Fig. 4O) and at the distal tip of the egg cylinder (*Noto*; Fig. S3H). No signals were observed anterior to the primitive streak. Thus, although mesoderm clearly arose in transgenic embryos (Fig. 4N), cells did not organize into PNC and notochordal plate during the course of gastrulation. Next, axis specification was analyzed, as *Gsc* acts as a potent inducer of secondary axes in *Xenopus*. Transcripts of *Otx2*, which marks the anterior pole (Fig. S3I), and *Fgf8*, which is expressed in the posterior part of the embryo (Fig. S3K), were found localized in the anterior and posterior half of the mutant egg cylinders as well (Fig. S3J,L). The AP-axis, therefore, was correctly specified in transgenic embryos, even in the most severe cases (Fig. S3J,L, and data not shown). Taken together, *Gsc* expression along the entire primitive streak of the mouse gastrula embryo impaired axial elongation, without affecting the patterning of embryonic tissues, and caused NTDs comparable to the BPDs and NTDs seen in *Xenopus*.

### Gsc inhibits Wnt/PCP

**Figure 5:**
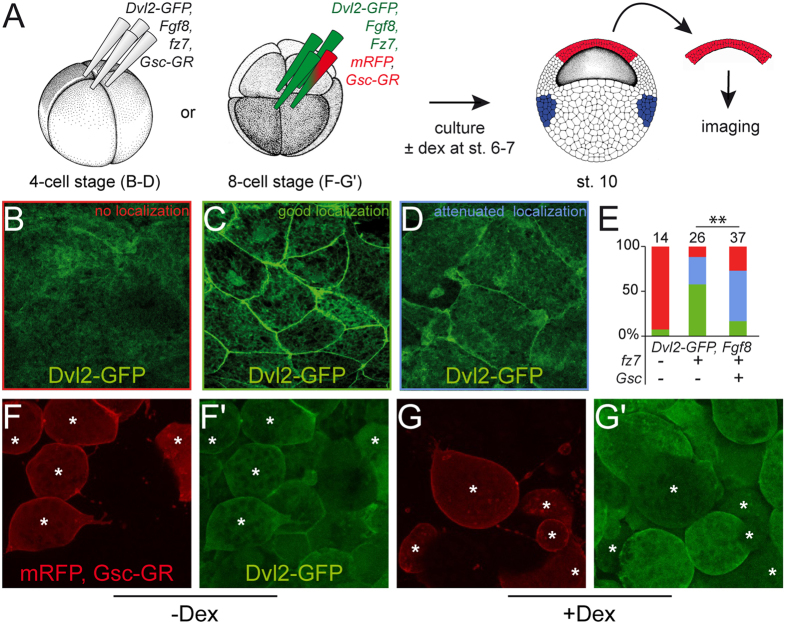
*Gsc-GR* inhibits membrane recruitment of Dvl2. (**A**) Co-injection of mRNAs as indicated into the animal region of all cells at the 4-cell stage or of selected cells at the 8-cell stage. Embryos were cultured ± dex (added at st. 6/7), animal cap tissues were excised at stage 10 and subjected to live imaging. (**B–E**) Membrane localization of Dvl2-GFP was significantly impaired upon Gsc-GR activation. (**B–D**) Examples of specimens from the same batch of embryos and photographed with the same exposure times showing lack of localization (**B**; red), good (**C**; green) and attenuated localization (**D**; blue). (**E**) Quantification of results (p = 0.002). (**F,G**) Cell-autonomous effect of Gsc-GR. Injection of *Gsc-GR* in 1/4 animal cap cells at the 8-cell stage (cf. **A**) resulted in attenuation of Dvl2-GFP membrane recruitment upon dex treatment (cf. **F’** and **G’**). *mark *Gsc-GR*-injected cells, as revealed by fluorescence of lineage tracer mRFP.

CE in frog and mouse is regulated by non-canonical Wnt signaling, specifically the PCP pathway^49^,^50^,^51^. One of the hallmarks of PCP signaling is the recruitment of Dvl2 to the plasma membrane^52^,^53^, which is compromised when PCP signaling is impaired^54^,^55^. We therefore wondered whether Gsc was able to interfere with Dvl2 localization. In *Xenopus*, a Dvl2-GFP fusion protein serves to investigate the subcellular localization in animal cap explant cultures^56^. Upon expression of the Wnt receptor Fz7, Dvl2-GFP translocated from the cytoplasm to the plasma membrane (Fig. 5C,E). Animal caps represent a naïve stem cell-like tissue that can be differentiated into descendants of all three germ layers^57^. As *Gsc* expression in the early vertebrate embryo is limited to mesodermal tissues^58^,^59^, animal cap explants were injected with the mesoderm-inducing isoform of *Fgf8*, Fgf8b, which was verified by germ layer-specific marker gene expression^60^ (Fig. S4). In order to assess whether Gsc impacted on Dvl2 subcellular localization, *Dvl2-GFP, fz7, fgf8* and *Gsc-GR* were coinjected into the animal region of 4–8 cell embryos, specimens were cultured in the presence or absence of dex until control embryos reached stage 10.5, when animal caps were excised and imaged (Fig. 5A). In the absence of dex, Dvl2-GFP relocated from the cytoplasm to the plasma membrane (Fig. 5B,E). When Gsc activity was induced following dex treatment, Dvl2-recruitment to the cell membrane was severely compromised (Fig. 5D,E-; p = 0.002). Gsc-GR acted in a cell-autonomous manner, as Dvl2 membrane localization was not affected in neighboring cells when *Gsc-GR* was only injected and activated in a subset of animal cap cells (Fig. 5F,G). These data demonstrated that in overexpression assays Gsc was clearly able to interfere with the recruitment of Dvl2 to the membrane as a prerequisite of non-canonical Wnt signaling and CE, in agreement with the observed gain-of-function phenotypes in mouse and frog.

### Wnt/PCP pathway components rescue Gsc-induced NTD/BPD

Our hypothesis that Gsc interferes with Wnt/PCP signaling predicted that pathway components should be able to rescue the *Gsc-GR* induced gain-of-function phenotypes NTD and BPD *in vivo*. The downstream effector *RhoA* was assessed, which regulates CE by reorganization of the actin cytoskeleton^61^. A constitutively active (ca) construct was used as well as a dominant-negative (dn) form of *RhoA* (Paterson *et al*.^90^). Both have been shown to induce BPD and NTD^61^, like most PCP components, which give rise to similar phenotypes upon gain- and loss-of-function^62^. In addition, the core PCP components *Vangl2* and *Prickle* were investigated, as they are required for subcellular localization of Dvl2^63^,^64^. In addition, the potential of *Wnt11* and *Xbra* to rescue Gsc-mediated phenotypes was analyzed, as both are known to induce CE in *Xenopus*^65^,^66^.

**Figure 6:**
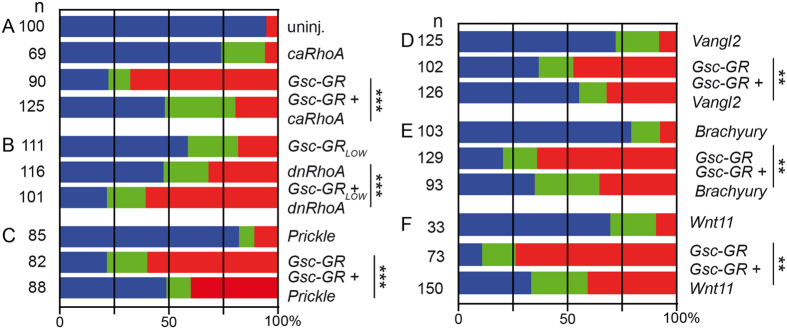
Rescue of Gsc-GR mediated NTD/BPD by Wnt/PCP pathway components. *Xenopus* embryos were injected with the indicated mRNAs into the dorsal marginal region of all cells at the 4-cell and cultured to stage 22. Dex was added when *Gsc-GR* was used. Specimens were scored for normal appearance (blue bars), NTD (green) and BPD (red). (**A**) constitutively active *RhoA*; (ca; **A**) dominant-negative (dn) *RhoA*; (**C**) *Prickle*; (**D**) *Vangl2*; (**E**) *Brachyury*; (**F**) *Wnt11*. Uninjected embryos (uninj.) served as controls. Note that rescue was observed upon co-injection of *Gsc-GR* with *ca-RhoA, Prickle, Vangl2, Brachyury* and *Wnt11*, while enhanced phenotypes were seen with co-injected *dn-RhoA*. As embryos in the latter combination showed high rates of lethality, the dose of injected *Gsc-GR* was reduced from 400 pg to 160 pg. Cf. Table S1 for numbers and statistics.

NTD and BPD were observed when *Gsc-GR* or any of the PCP components were injected into the dorsal marginal zone (Fig. 6). To test if and how Gsc interacted with PCP signaling, co-injection experiments were performed. *caRhoA* significantly decreased the percentage of malformed embryos induced by *Gsc-GR* (Fig. 6A; Table S1). In order to analyze whether *dnRhoA* enhanced the Gsc effects accordingly, both were co-expressed. High lethality of embryos prevented the quantitative analysis of the experiment (not shown). When the dosage of the injected *Gsc-GR* construct was lowered 2.5-fold, *dnRhoA* co-injection resulted in a significantly higher percentage of affected specimens as compared to the injection of *dnRhoA* alone (Fig. 6B; Table S1). As RhoA is a general modifier of actin cytoskeleton dynamics, we extended our study to core PCP pathway components. Co-injections of *Prickle* and *Vangl2* partially rescued the Gsc-induced phenotypes (Fig. 6C,D; Table S1). In addition, mouse *Brachyury* and *Xenopus Wnt11* were also able to partially revert Gsc-GR induced NTD and BPD (Fig. 6E,F; Table S1). In summary, these gain-of-function experiments demonstrated the potential of Gsc to act as a negative regulator of PCP-mediated CE, at least in the context of gain-of-function induced phenotypes.

### Wnt/PCP phenotypes in *Gsc* morphant frog and mutant mouse embryos

**Figure 7:**
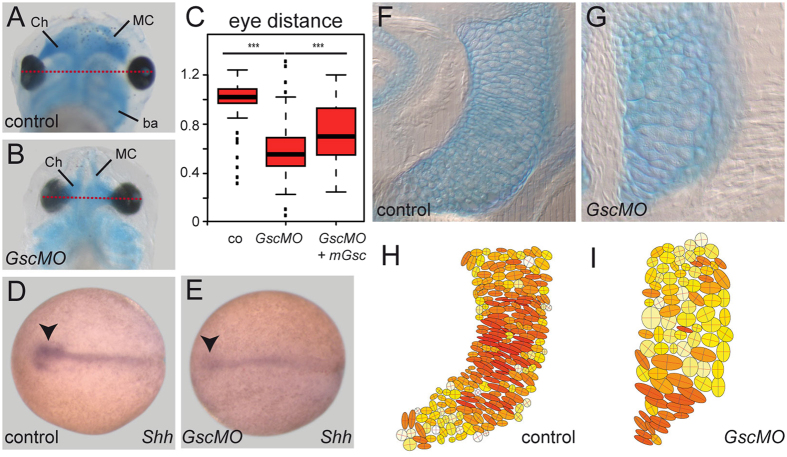
Prechordal plate and cartilage defects in *Gsc* morphant *Xenopus* tadpoles. (**A–E**) Prechordal plate defects. (**A**–**C**) Close-set eyes in *Gsc* morphants. Distance between left and right eye (red lines) was reduced in morphants. Arithmetic mean of control specimens was set to 1.0 in (**C**). Note that this phenotype was rescued by co-injection of a mouse *Gsc* cDNA construct. (**D,E**) *Shh* mRNA expression in control (**D**) and high dose *Gsc* morphant (**E**). Note that the prechordal plate (arrowheads) was severely reduced in morphants. (**F–I**) Cartilage phenotypes in *Gsc* morphant frog tadpoles. Cartilage was stained with alcian blue in wt (**F,H**) or *Gsc* morphant (**G,I**) tadpoles at stage 45. Shape of cartilage cells of was analyzed in frontal sections of embryos (**F,G**). (**H,I**) Cells were outlined with ImageJ and aspect ratios were calculated and visualized. Cell shapes are indicated by a color gradient from yellow to red, with round cells depicted in light yellow and elongated bipolar cells in deep red. Note that the majority of cartilage cells in *Gsc* morphants had lost their bipolar appearance.

In order to analyze whether the endogenous Gsc is involved in inhibition of Wnt/PCP-mediated CE as well, we re-investigated *Gsc* morphant frog embryos and knockout mouse specimens. In *Xenopus* we used a previously characterized *Gsc* MO^21^. Analysis of morphant tadpoles revealed that the eye distance was significantly reduced at stage 45 compared to uninjected control specimens (Fig. 7A,B). Co-injection of a full-length mouse *Gsc* cDNA construct, which was not targeted by the MO, partially rescued this phenotype, demonstrating the specificity of the MO (Fig. 7C). As during development the eye field is split by the prechordal plate, which expresses *Gsc*, we hypothesized that this population of migrating cells was affected in morphants. *Shh* mRNA transcription was analyzed, which along the axial midline is expressed in the prechordal plate mesoderm and the floorplate of the neural tube. Figure 7(D,E) shows that the width of the anteriormost *Shh* expression domain, i.e. the expression in or above the prechordal plate, was narrowed, in line with the observed close-set eyes.

To analyze whether the notochord was expanded at the expense of the prechordal plate, which was previously suggested in experiments using antisense *Gsc* DNA expression constructs^16^, *Xbra* mRNA expression was investigated in morphant specimens. Surprisingly, the notochord appeared wider and shorter, as compared to wt specimen (Fig. S5). The aspect ratio, which was set to 1.0 in control specimens, was significantly reduced to 0.61 in morphants (Fig. S5C). As we had noted this particular phenotype in *Gsc* gain-of-function specimens (Fig. 1F–I), we wondered whether *Gsc* transcription was affected in *Gsc* morphants. The *Gsc* expression domain in morphants was indeed stronger and expanded both laterally and posteriorly towards the blastopore (Fig. S5G,H). This at first glance paradoxical finding, however, is in good agreement with our previous finding of a negative auto-regulatory feedback loop of Gsc on its own transcription^67^. The analysis of MO-mediated *Gsc* loss-of-function phenotypes thus might be hampered by limiting MO-doses, which might be insufficient to prevent the translation of additional transcripts generated by the release of the negative autoregulatory *Gsc* feedback loop. When the MO doses were increased to counteract this possible effect, the length of the notochord was slightly expanded to an aspect ratio of 1.14 in morphants (p = 0.0193), an effect which was partially (and non-significantly) reversed by co-injection of the mouse rescue cDNA construct (aspect ratio 1.07; Fig. S5D–F). These tendencies may suggest that MO doses have, indeed, been limiting.

In addition to a reduced eye distance we noted that the morphology of the head cartilage was altered in *Gsc* morphant tadpoles at stage 45, in particular Meckel’s cartilage and the ceratohyale (Fig. 7A,B,F–I). In mouse, *Gsc* is expressed in undifferentiated branchial arch mesenchyme and persists as these tissues undergo differentiation into head cartilage^68^. Re-evaluating *Gsc* expression during late tadpole development revealed a like expression pattern in *Xenopus* as well (Fig. S6). As cartilage condensation involves CE^69^,^70^, we wondered whether morphological alterations in morphants were reminiscent of PCP phenotypes. To that end we analyzed cellular morphologies of cartilage cells. While wt cells displayed predominantly bipolar morphologies (Fig. 7F,H), evaluation of length vs. width aspect ratios demonstrated loss of elongated cell shapes in morphants (Fig. 7G,I). This phenotype strikingly resembled the failure of Meckel’s cartilage cells to elongate and intercalate in morphants of the PCP effectors *inturned* and *fuzzy*^70^, suggesting that the cartilage phenotype in *Gsc* morphant tadpoles represented a PCP-phenotype as well.

**Figure 8:**
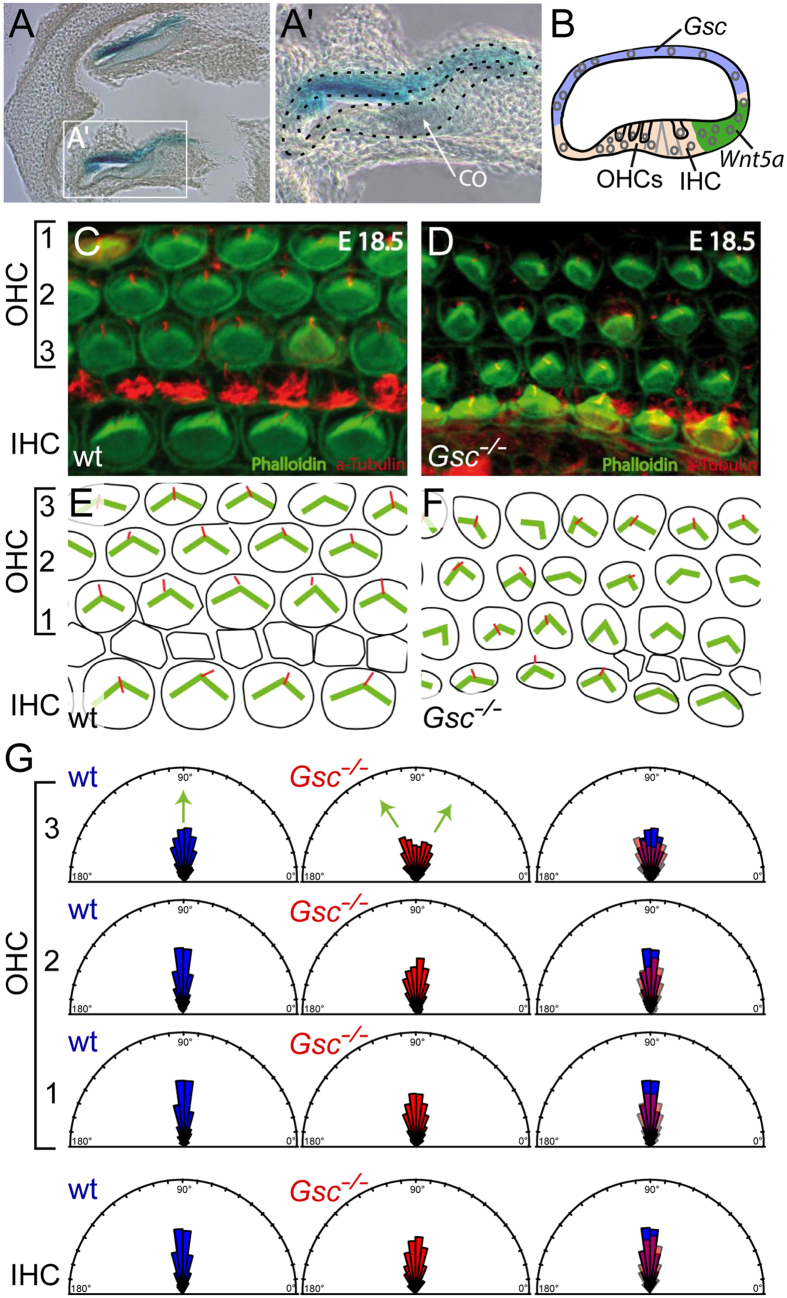
Disrupted alignment of outer hair cells (OHCs) in the cortical organ of *Gsc* knockout embryos. (**A**) *Gsc* transcription (blue) locates opposite of the cortical organ (CO). (**B**) Schematic depiction of *Gsc* expression in blue and *Wnt5a* expression in green. OHCs and inner hair cells (IHC) are highlighted by arrows. (**C–G**) Confocal imaging of kinocilia (red, tubulin) and stereocilia (green, phalloidin) in the cortical organ of *Gsc* knockout mouse embryos (**D,F**), compared to wt littermates (**C,E**) schematically depicted in (**E,F**). (**G**) Quantification of alignments, depicted as rose plots. According to the angle of deviations from the normal perpendicular orientation (90°), vectors were plotted in 11.25° sectors. The area of a sector represents the number of cells with this directionality. Note that significantly higher deviations from the normal perpendicular orientation (90°) were observed in OHC3 of *Gsc* knockout specimens (middle, red, n = 390) compared to wildtype littermates (left, blue, n = 308, p = 0.03).

Finally, we re-investigated *Gsc*-knockout mouse embryos for potential PCP/CE phenotypes. Besides the above-mentioned expression around condensing cartilage, the inner ear is the organ that has been particularly well characterized with respect to PCP in the mouse. As previously described, *Gsc* was expressed in the inner ear opposite the organ of Corti^71^ (Fig. 8A,B), and opposite the expression domain of the non-canonical Wnt ligand *Wnt5a*^72^ (Fig. 8B). Stereo- and kinocilia of outer and inner hair cells (OHC/IHC) display a distinctive planar cell polarity and are a well-known target of PCP-signaling^73^. To investigate whether PCP of inner ear hair cells was altered in *Gsc* knockout embryos, E18.5 cochleas were isolated from wt and knockout specimens and analyzed for stereo- and kinocilia orientation. Phallodin staining was used to highlight the actin cytoskeleton of the V-shaped stereocilia, and tubulin staining to visualize the axoneme of the kinocilium. In wt and heterozygous E18.5 specimens, stereo- and kinocilia of IHCs and OHCs align and point towards the periphery of the cochlea (Fig. 8C,E). In *Gsc* knock-out embryos, however, this orientation was disrupted (Fig. 8D,F). A quantification of average deviations from the normal perpendicular orientation revealed higher values in *Gsc* knockout specimens, which was significantly pronounced in outer hair cell row 3 (Fig. 8G, p = 0.03, n = 390) compared to wt littermates (n = 308). This result unequivocally demonstrated that *Gsc* knockout mouse embryos displayed a well-characterized Wnt/PCP phenotype as well. Taken together, our *Gsc* gain- and loss-of-function studies in frog and mouse embryos revealed a novel role of Gsc as an inhibitor of Wnt/PCP-mediated cell morphogenesis and behavior, in particular CE.

## Discussion

A quarter of a century ago, the first description of *Gsc*’s potential to induce secondary axis formation set the starting point for an extremely productive molecular analysis of Spemann’s organizer^7^. The apparent lack of gastrulation phenotypes in mutants and morphants reduced the perceived relevance of *Gsc* to being the best available marker of organizer tissue across the animal kingdom. Our present report of a novel function of *Gsc* as transcriptional inhibitor of Wnt/PCP-mediated CE not only offers a potential mechanism to understanding the various malformations of bone and cartilage in *Gsc* knockout mice (and human patients^74^). It may as well assign a role for *Gsc* in the organizer-derived prechordal plate, namely to restrict CE to the notochord and to facilitate or enable the migration of the prechordal mesodermal cells. Our conditional gain-of-function analyses in frog and mouse clearly demonstrate the potential of Gsc to act as an inhibitor of Wnt/PCP-mediated CE. The analysis of loss-of-function phenotypes in both model systems supports such a role during embryonic development, although - admittedly - they represent in parts initial and preliminary characterizations. A key question, that remains unanswered, relates to the molecular mechanism of Gsc function in inhibiting Wnt/PCP. Two aspects, which our experiments touch upon, deserve further elaboration, namely whether this effect is cell- or non-cell autonomous and how novel target genes were recruited under the control of Gsc.

As mentioned in passing, it is not possible to target the axial mesoderm/notochord in *Xenopus* without at the same time delivering constructs to the floorplate of the neural tube. Thus, the observed NTDs could represent a cell-autonomous effect of ectopic Gsc expression. The cell-autonomous interference of Gsc-GR with Dvl2 membrane recruitment in animal caps (cf. Fig. 5F,G) supports this notion. In the conditional mouse experiments, however, ectopic *Gsc* expression was strictly limited to the primitive streak mesoderm, as the *Brachyury* streak enhancer is only active there^46^. NTDs in mouse, therefore, cannot be caused by a cell-autonomous Gsc function. The same reasoning holds true for the inner ear: here *Gsc* is expressed opposite to the IHCs/OHCs at the organ of Corti that undergo PCP. Further, *Gsc* and the Wnt ligand Wnt5a, which has been shown to be the decisive ligand for the arrangement of these cells^75^, are expressed in adjacent rather than the same cells, demonstrating that the inner ear phenotype in the genetic knockout situation is the result of a non-cell autonomous effect of Gsc. It thus appears that context-dependently Gsc acts in a cell or non-cell autonomous manner to repress PCP/CE.

**Figure 9:**
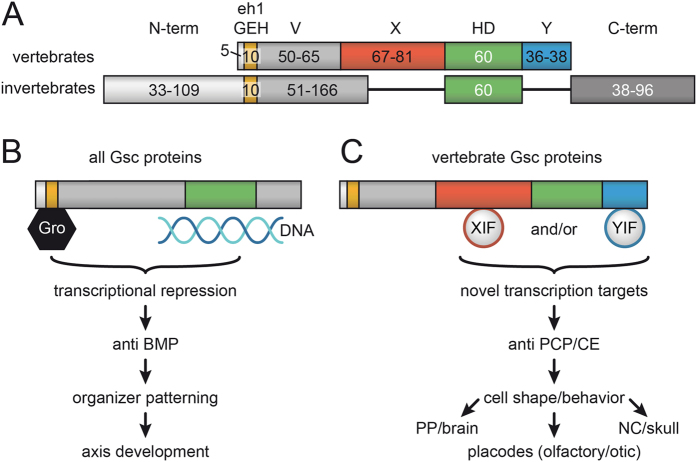
Vertebrate-specific Gsc functions: a model. (**A**) Domain structure of invertebrate and vertebrate Gsc proteins; numbers indicate ranges of amino acids. The engrailed homology (eh1/GEF) repression domain and the homeodomain (HD) are common to all Gsc proteins. Two highly conserved domains (X, Y) flanking the HD emerged at the base of the vertebrates. Note that invertebrates, besides lacking X/Y, possess variable length N- and C-terminal sequences and that the linker region between eh1/GEF and HD also varies greatly in length. (**B**) All Gsc proteins have the potential to act as transcriptional repressors through HD-binding to DNA and Groucho-recruitment to eh1/GEF. When assayed in *Xenopus, Drosophila* and vertebrate Gsc proteins act in organizer patterning and axis development through their conserved anti-BMP function. (**C**) Vertebrate Gsc proteins in addition affect cell shape and behavior through their anti PCP/CE function. We propose that X- and Y-domain interacting factors XIF and YIF function in recruiting novel transcriptional target genes under Gsc control. We further propose that this novel function of Gsc co-evolved with the vertebrate-specific novelties of an enlarged brain, skull and placodes, as vertebrate *Gsc* is expressed in the prechordal plate/floor plate of the diencephalon, neural crest mesenchyme and derivatives as well as otic vesicle/nasal cavity.

The inability of *Drosophila Gsc* to interfere with PCP/CE (while inducing double axis formation even more efficiently than *Xenopus* or mouse *Gsc*) indicates that this function either arose during vertebrate evolution or was lost in *Drosophila*. To approach this question, we compared Gsc protein sequences across the animal kingdom. In invertebrates, no conserved regions besides the highly conserved eh1/GEH domain and a basically invariant homeodomain (HD) were found, arguing against a loss of anti-PCP/CE activity in *Drosophila* (Fig. 9A, Fig. S7). The presence of eh1/GEH and HD in all Gsc sequences in addition suggests that all proteins should have the potential to act as transcriptional repressors in organizer patterning and axis development, at least when assayed in *Xenopus*, a function which is mediated through Gsc’s well-documented anti-BMP function (Fig. 9B)^16^,^76^. Vertebrate Gsc proteins in contrast possess two novel highly conserved domains flanking the HD, which we address as “X” and “Y” (Fig. 9A, Fig. S7). Interestingly, both domains are absent in the cephalochordate amphioxus, in which neither a cranium nor neural crest have evolved yet^77^,^78^,^79^, as well as in the lamprey, a primitive agnathan vertebrate that has neural crest but lacks jaws^80^ (not shown). When databases were screened for sequences related to X and Y, exclusively vertebrate Gsc sequences were picked up (not shown). These data indicate that the anti-PCP/CE function evolved at the base of the vertebrates, likely together with the acquisition of domains X and/or Y (or parts thereof). We like to propose that X- and/or Y-interacting factors (XIF and YIF in Fig. 8C) recruited Gsc to novel target promoters, either by direct DNA-binding of XIF/YIF or through interaction with other DNA-binding proteins. Vertebrate-specific target genes could function directly upstream of PCP components. Alternatively, they may act in a parallel pathway that controls competence for Wnt/PCP signaling. Elucidating the molecular mechanisms will involve the identification of (1) target genes; (2) peptides mediating the vertebrate anti-PCP/CE function, for example by introducing X/Y sequences and fragments thereof into *Drosophila* Gsc and assaying recombinant genes in *Xenopus*; (3) XIF/YIF, for example through the identification of the interactome of identified peptides.

Relating the emergence of the anti-PCP/CE function at the base of the vertebrates to post-gastrulation expression patterns in the vertebrates reveals a potentially highly relevant coincidence: *Gsc* transcripts are found in (1) the prechordal plate and floor plate of the diencephalon; (2) branchial arch mesenchyme and derivatives (skull cartilage, tongue, etc.^68^); (3) placodal derivatives (otic vesicle/organ of Corti, olfactory pit/nasal passage^71^, i.e. in tissues representing evolutionary novelties of the vertebrates^81^. It is tempting to speculate that *Gsc* was recruited into gene regulatory networks specific to these tissues to shape their morphogenesis by regulating cellular morphology and behavior.

In the light of this reasoning, an in-depth re-evaluation of the endogenous Gsc functions in the various vertebrates is in demand. While this manuscript was under review, two relevant studies were published. The analysis of otic vesicle differentiation in zebrafish morphants and TALEN-induced mutants revealed a function for *Gsc* in the delamination of neuroblasts, i.e. a process involving epithelial-to-mesenchymal transitions associated with cell shape changes and delamination/migration behavior^82^. Inner ear hair cell PCP was not investigated in this study. In *Xenopus*, aCRISPR/Cas9 approach to genome-editing of - among others - *Gsc* was reported and specimens were shown to display massive head defects, that were not further characterized^83^ but in perfect agreement with the neural crest/skull phenotypes reported here. Genome editing should provide a powerful complementing means to the use of MOs for studying *Gsc* function, as applicable MO-doses may be the limiting factor in such experiments, based on the observed gain-of-function by loss-of-function, i.e. interference with the negative auto-regulatory feedback loop (cf. Fig. S5G,H). Even antisense RNA may prove useful in the future. The late Herbert Steinbeißer and colleagues previously injected such RNAs into the axial midline and noted that the notochord was expanded at the expense of the prechordal plate^16^. Unfortunately, this loss-of-function approach fell in disgrace^84^ and the prechordal plate phenotype was never fully characterized.

The knockout mouse in any case deserves to be re-evaluated. When we analyzed *Gsc* expression domains during organogenesis stages, we found transcripts adjacent to tissues that elongate during development, which might involve PCP-mediated convergent extension. *Gsc* mRNA was for example found at the anterior tip of the tongue, in the arytenoid swellings and the palatal shelves^71^ (Fig. S8). The previously described limb bud expression fits to this proposal as well, as limb bud differentiation was identified as a PCP-dependent process as well^85^,^86^.

Finally, the early embryonic expression pattern of *Gsc* in vertebrate embryos is in agreement with such a function. The first transcription in the organizer tissue itself is very transient. As axial mesodermal cells (prechordal plate and notochord) migrate out in rostral direction, *Gsc* is downregulated in the organizer, maintained in the prechordal cells and absent in the notochord^10^,^26^,^27^. Segregation of organizer-derived cells into these two populations is accompanied by differences in cell behavior (single cell migration of the prechordal cells and CE in the notochord) and gene expression (*Gsc* in the prechordal and *Brachyury* in the notochordal mesoderm^28^,^29^,^30^,^87^,^88^). Gsc, thus, may provide the switch between cell intercalation and cell migration by limiting CE to the notochord. 25 years after the first characterization of *Gsc* in the organizer, the fascination for this gene continues. Much has to be learned about its function in development and disease.

## Methods

All methods were performed in accordance with the relevant guidelines and regulations.

### Statement of approval of animal experimentation

Handling, care and experimental manipulations of were approved by the Regional Government Stuttgart, Germany (Vorhaben A379/12 ZO “Molekulare Embryologie”), according to German regulations and laws (§6, article 1, sentence 2, nr. 4 of the animal protection act).

### Plasmids and construction of Xenopus expression vectors

K197E^17^ was obtained from Dan Kessler, Wnt11 constructs were from Kristen Kwan, Vangl2 from Ray Keller, and Prickle1 from Naoto Ueno. Gsc-GR has been described in ref. 89. Fgf8, Fz7, Dvl2-GFP, dnRhoA and caRhoA constructs were provided by the Steinbeißer laboratory.

The following PCR primers were used for cloning of deletion constructs ∆HD and ∆GEH: ∆HDfor 5′-ATATCGATGCGCTGCAAGGAGTCGCTGCTG-3′, ∆HDrev 5′-CTGGACTCTGACAGTGGTCCTCGAGAT-3′, ∆GEHfor 5′-ATATCGATGCGCTGCAAGGAGTCGCTGCTG-3′, ∆GEHrev 5′-CTGGACTCTGACAGTGGTCCTCGAGAT-3′. The starting construct to clone T-Gsc was PML129 (vector backbone PGEM3, Promega), which contained the 658 bp Brachyury streak promoter, followed by a floxed LacZ cassette with triplicate polyadenylation signals to ensure that the downstream open reading frame is not part of the mRNA. To create construct T-Gsc the 771 bp Gsc coding sequence was inserted downstream, flanked by a 231 bp polyadenylation signal from the bovine growth hormone gene (from pRc/CMV, Invitrogen). Construct mT-Gsc was generated by mutating the Brachyury streak promoter 35 bp downstream of the transcriptional start site from TAAT into ACTG^11^.

### Generation of transient chimeric embryos

Two constructs were used to transfect mouse ES cells (line E14-KPA, kindly provided by Klaus Peter Knobeloch, FMP, Berlin, Germany), a Gsc and a LacZ expression construct, which both used the human ubiquitin promoter. Stable lines were selected by co-transfection of the selection plasmid containing the PGK-neo cassette. Individual clones were characterized for transgene expression by RT-PCR analysis (pcubi-Gsc primer; see below). A clone displaying high expression levels was used in blastocyst injection experiments to derive transgenic embryos which were harvested at E9.5 and E10.5.

### Generation of T-Gsc and mT-Gsc mouse lines and Cre-mediated transgene activation

Inserts of vectors were isolated by KpnI enzyme digestion and introduced by electroporation into E14-KPA and CJ7 cells (kindly provided by Thomas Gridley, Jackson Laboratory, USA), and cultured following standard procedures. After G418 selection (250 μg/ml), four transgenic clones were identified with T-Gsc and 28 clones with mT-Gsc, each containing single copy gene integration verified by Southern blot analysis. Reporter gene activity was tested by X-gal staining of mesodermally differentiated clones, which express *Brachyury*. Mesodermal differentiation was performed in hanging drop cultures in the presence of DMSO. Clones showing strong reporter gene activity were used to generate transgenic mice, which were derived from C57BL/6 J blastocyst injections. Offspring of germ line-transmitting chimeric mice were screened for the presence of the T-Gsc transgene. Heterozygous mice were kept on a mixed background and mated to obtain homozygous animals. One line was obtained with T-Gsc and two lines with mT-Gsc. Transgenes were activated by crossing homozygous deleter females with homozygous T-Gsc or mT-Gsc males.

### Genotyping of transgenic mice and embryos

DNA from embryos and tail biopsies was isolated using standard protocols. Primers and PCR conditions were as follows:

LacZ primer: a) 5′-TCAATCCGCCGTTTGTTCC; 3′-CCGCCACATATCCTGATCTTCC; 280 bp, 55 °C b) 5′-GCAGTGCACGGCAGATACACACTT; 3′-CCCCATATGGAAACCGTCG; 510 bp. 55 °C; c) 5′-GGGACGCGCGAATTGAATTGAATTA; 3′-CCCCATATGGAAACCGTCG; 160 bp, 55 °C;

Cre primer: a) 5′-CGCATAACCAGTGAAACAGCAT; 3′-GAAAGTCGAGTAGGCGTGTACG; 550 bp, 55 °C b) 5′-TAATCGCCATCTTCCAGCAG; 3′-GCTGGCTGGTGGCAGATGGCG; 650 bp, 55 °C; c) 5′-CAATTTACTGACCGTACAC; 3′-GCTGGCTGGTGGCAGATGGCG; 751 bp, 55 °C; Gsc-bGHpA primer: 5′-GTTCTGTACTGGTGTCTCG (in Exon3 of Gsc); 3′-GGCACCTTCCAGGGTCAAGG (in the polyadenylation signal of the bovine growth hormone); 277 bp, 63.5 °C; pcubi-Gsc 5′-CCACTAGTCCAGTGTGGTGG; 3′-GACGCAGGGCTGCGGGGGTC; 385 bp, 65 °C.

### Manipulations of Xenopus embryos

For microinjections, drop size was calibrated to about 8 nl/injection. Embryo culture and microinjection followed standard procedures. mRNAs were prepared using the Ambion message machine kit. DsRed mRNA (1.6 ng mRNA/embryo) and rhodamine-B dextran (0.5–1.0 μg/μl; Molecular Probes) were used as lineage tracers. Unless indicated otherwise, 400 pg Xgsc-GR mRNA/embryo was injected^32^. Gsc-GR fusion protein was activated by the addition of 10 μg/ml dexamethasone at stage 6–8 (unless specified otherwise). Concentrations of injected mRNAs (transcribed from CS2^+^-expression vectors) were: constitutive active RhoA V14 (32–64 pg mRNA/embryo), dominant negative RhoA N19^90^ (320 pg mRNA/embryo), *Prickle1*^91^ (1.8 ng/embryo), *Vangl2/Strb*^63^ (400 pg/embryo), *T* (800 pg mRNA/embryo; cds of mouse Brachyury), and *Wnt11*^29^ (80 pg mRNA/embryo). For Knock-down experiments a coding morpholino was used (5′-GCTGAACATGCCAGAAGGCATCACC-3, Gene Tools LLC^21^. Statistical calculations were performed using Pearson’s chi-square test comparing the number of affected embryos against the number of wt embryos (Statpages.com).

### Manipulations of Xenopus explants

Keller open face explants were prepared as described^37^,^87^, except that DFA medium was used. Animal cap assays were conducted according to Green, 1999. All cells of the 4-cell embryo were injected into the animal pole, dex was added at stage 6, where indicated, and the animal caps were cut at stage 9. Recombinant human Activin A (R&D Systems) was added immediately after cutting and the embryos were cultured until control specimens reached stage 22–30. For the Dvl2 localization assay, the following mRNAs, transcribed with the Ambion message machine from CS^2+^ vectors, were injected: a construct containing the C-terminal DEP-domain of Dvl2 fused to GFP (400 pg/cell; D9^56^), *Frizzled7*^92^ (400 pg/cell), *Fgf8* (8.8 pg/cell), *Gsc-GR*^32^ (560 pg/cell). Explants were cultured until control siblings reached stage 10.5.

### RT-PCR

Total RNA was isolated from animal cap explants at stage 10.5, and cDNAs were prepared using standard protocols. Primers used for amplification where from different exons to avoid genomic contamination. *EF1alpha* served as loading control. EF1α: for 5′-ACTGCCTTGATGATGACTCCTAG rev 5′-CAGATTGGTGCTGGATATGC; *Wnt11*: for 5-TGACGGTCTAGTCCCTGACCA, rev 5′-GGTTGCAGCTGTCACCTACCA; *Xbra:* for 5′-CACAGTTCATAGCAGTGACCG, rev 5′-TTCTGTGAGTGTACGGACTGG.

### Analysis of cell proliferation and apoptosis

Immunofluorescence was performed on whole-mount embryos, fixed for 1–2 hours at room temperature in 4% PFA for cell proliferation or in methanol/DMSO (4:1; Dent’s solution) for assessment of apoptosis. Embryos were processed as previously published and according to standard procedures^93^,^94^. Ethanol treatment (2.5%) served as positive control for the apoptosis assay. Primary antibodies: polyclonal rabbit anti-phospho-Histone H3 (Ser10; 1:700; Merck), monoclonal rabbit anti-caspase-3 Ab (1:150; 9665, Cell Signaling Technologies). Secondary antibody: Alexa Fluor 488-conjugated goat anti-rabbit (1:750, Invitrogen).

### RNA *in situ* hybridization and histological analysis

*Xenopus* and mouse embryos were fixed in 4% PFA for 2 hrs and processed following standard protocols. Digoxigenin-labelled (Roche) RNA probes were prepared from linearized plasmids using SP6 or T7 RNA polymerase (Promega). *In situ* hybridization was performed as described^95^. Cartilage was stained with 0.05% alcian blue followed by bleaching. For histological analysis embryos were embedded in gelatine-albumin and sectioned on a vibratome (30 μm).

### Analysis of cell shape and gene expression domains

Aspect ratios of cell shape and gene expression domains as well as statistical significances were calculated by Mann-Whitney-U test in statistical R (R-Development-Core-Team, 2008). The whiskers of the box plots extend to maximal 1.5 × IQR, outliers are displayed as dots. Aspect ratio = major axis/minor axis. Major and minor are the primary and secondary axis of the best fitting ellipse.

### Scanning Electron Microscopy

SEM analysis was performed following published protocols^96^. In brief, embryos were dissected and immediately fixed in 2.5% glutaraldehyde in Soerensen’s buffer (0.1 M sodium phosphate buffer; pH 7.4). Specimens were postfixed in 1% OsO_4_, critical point dried, sputter coated, and examined using a Zeiss DSM 940 A SEM (Oberkochen, Germany).

### Analysis of the cortical organ

The inner ear of E18.5 embryos was dissected and fixed in 4% PFA for 2 days at 4 °C. Cochleae were opened for better accessibility and stained with a mouse monoclonal antibody directed against acetylated alpha tubulin (1:700; Sigma), Cy3-conjugated secondary polyclonal rabbit sheep anti mouse antibodies (Sigma; 1:250) and Alexa Fluor^®^ 488 Phalloidin (Molecular probes, 1:40) following standard procedures, and imaged using a Zeiss LSM Pascal 5 Confocal Laser Scanning Microscope.

To determine stereociliary bundle orientation, we used the angle measurement tool in ImageJ, measuring the angle between the line from the position of the kinocilium through the middle of the “V”-shaped stereocilia and a line parallel to the mediolateral axis. In perfectly aligned cells, this angle is 90°. A Wilcoxon rank sum test with continuity correction in statistical R (R-Development-Core-Team, 2008) was used for statistical analyses.

## Additional Information

**How to cite this article:** Ulmer, B. *et al*. A novel role of the organizer gene *Goosecoid* as an inhibitor of Wnt/PCP-mediated convergent extension in *Xenopus* and mouse. *Sci. Rep.* **7**, 43010; doi: 10.1038/srep43010 (2017).

**Publisher's note:** Springer Nature remains neutral with regard to jurisdictional claims in published maps and institutional affiliations.

## Supplementary Material

Supplementary Information
