# Supplementary material for: A novel role of the organizer gene Goosecoid as an inhibitor of Wnt/PCP-mediated convergent extension in Xenopus and mouse
Source: Sci Rep. 2017 Feb 21;7:43010. doi: 10.1038/srep43010 (PMC5318956; doi:10.1038/srep43010)
Supplement: Supplementary Information [file srep43010-s1.pdf]

Supplementary Information

A novel role of the organizer gene *Goosecoid* as an inhibitor of Wnt/PCP-mediated convergent extension in *Xenopus* and mouse

Bärbel Ulmer[1§+], Melanie Tingler[1§], Sabrina Kurz[1§], Markus Maerker[1§], Philipp Andre[1], Dina Mönch[1], Marina Campione[1*], Kirsten Deißler[1], Mark Lewandoski[2], Thomas Thumberger[1&], Axel Schweickert[1], Abraham Fainsod[3], Herbert Steinbeißer[4] and Martin Blum[1#]

[1]University of Hohenheim, Garbenstr. 30, 70599 Stuttgart, Germany

[2]Genetics of Vertebrate Development Section, Cancer and Developmental Biology Lab, National Cancer Institute, National Institutes of Health, Frederick, MD 21702, USA

[3]Department of Developmental Biology and Cancer Research, Institute for Medical Research Israel-Canada, Hebrew University, Jerusalem 9112102, Israel

[4]Institute of Human Genetics, University Hospital Heidelberg, Im Neuenheimer Feld 366, 69120 Heidelberg, Germany

[+]Present address: Department of Experimental Pharmacology and Toxicology, Cardiovascular Research Center, University Medical Center Hamburg-Eppendorf, 20246 Hamburg, Germany.

*Present address: CNR-Neuroscience Institute, Department of Biomedical Sciences, University of Padova, Italy

[&]Present address: Centre for Organismal Studies (COS) Heidelberg, Heidelberg University, Im Neuenheimer Feld 230, 69120 Heidelberg, Germany

[§]These authors contributed equally to the present work

[#]Corresponding author: martin.blum@uni-hohenheim.de

SUPPLEMENTARY INFORMATION

Supplemental Figures and Tables

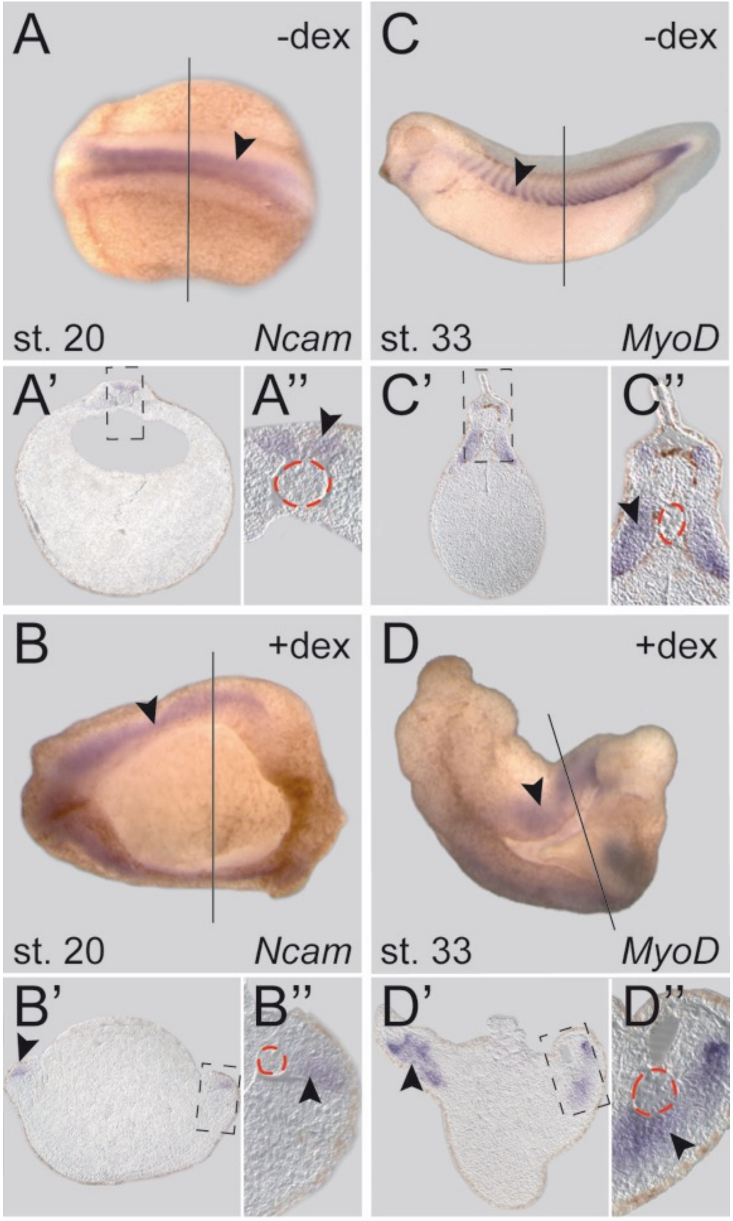

Fig. S1. Characterization of *Gsc-GR* mediated CE phenotypes in *Xenopus* embryos. Analysis of neural (*Ncam*; A, B) and paraxial mesodermal (somite; *MyoD*; C, D) marker gene expression in wildtype (-dex; A, C) and Gsc-GR expressing (+dex; B, D) embryos. Dex was added at stage 6-9, and embryos were analyzed for marker gene expression by whole-mount in situ hybridization following fixation at the stages indicated. Note that specification of examined tissues (arrowheads) was not affected. Solid lines indicate planes of sections, dashed boxes mark regions shown in higher magnification. Red dashed lines outline notochord.

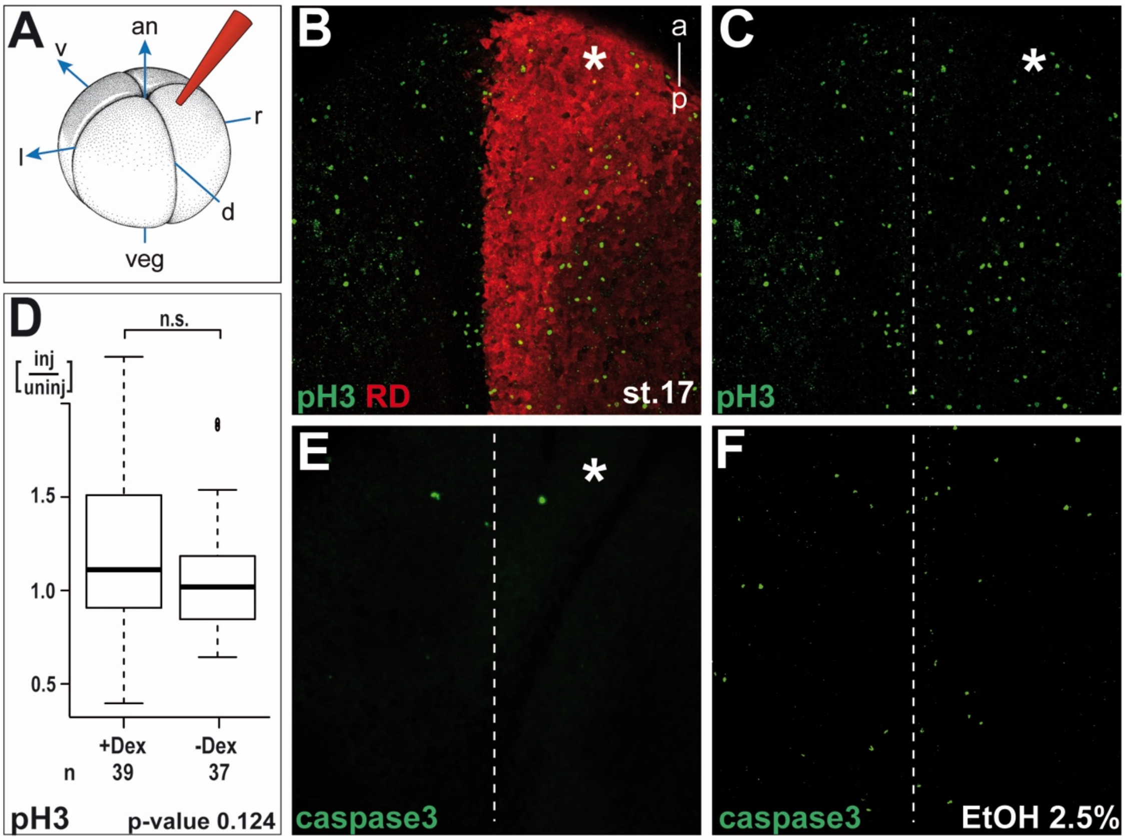

Fig. S2. Proliferation (A-D) and apoptosis (A, E, F) were unaffected upon Gsc-GR activation in the neuroectoderm. Embryos were unilaterally injected with *Gsc-GR* and lineage tracer rhodamine dextrane into the neuroectodermal (dorsal-animal) lineage at the 4-cell stage, dex was added (+Dex) or omitted (-Dex) between st.6-8, and specimens were cultured until stage 17. *, injected side. Proliferation and apoptosis were assessed by IF using an anti-pH3 (B, C) and anti-caspase3 antibodies (E, F), respectively. (D) Evaluation of proliferation. (F) Ethanol treatment at stage 13 (2.5%) served as a positive control for induction of apoptosis. Note that neither proliferation nor apoptosis were affected by Gsc-GR activation.

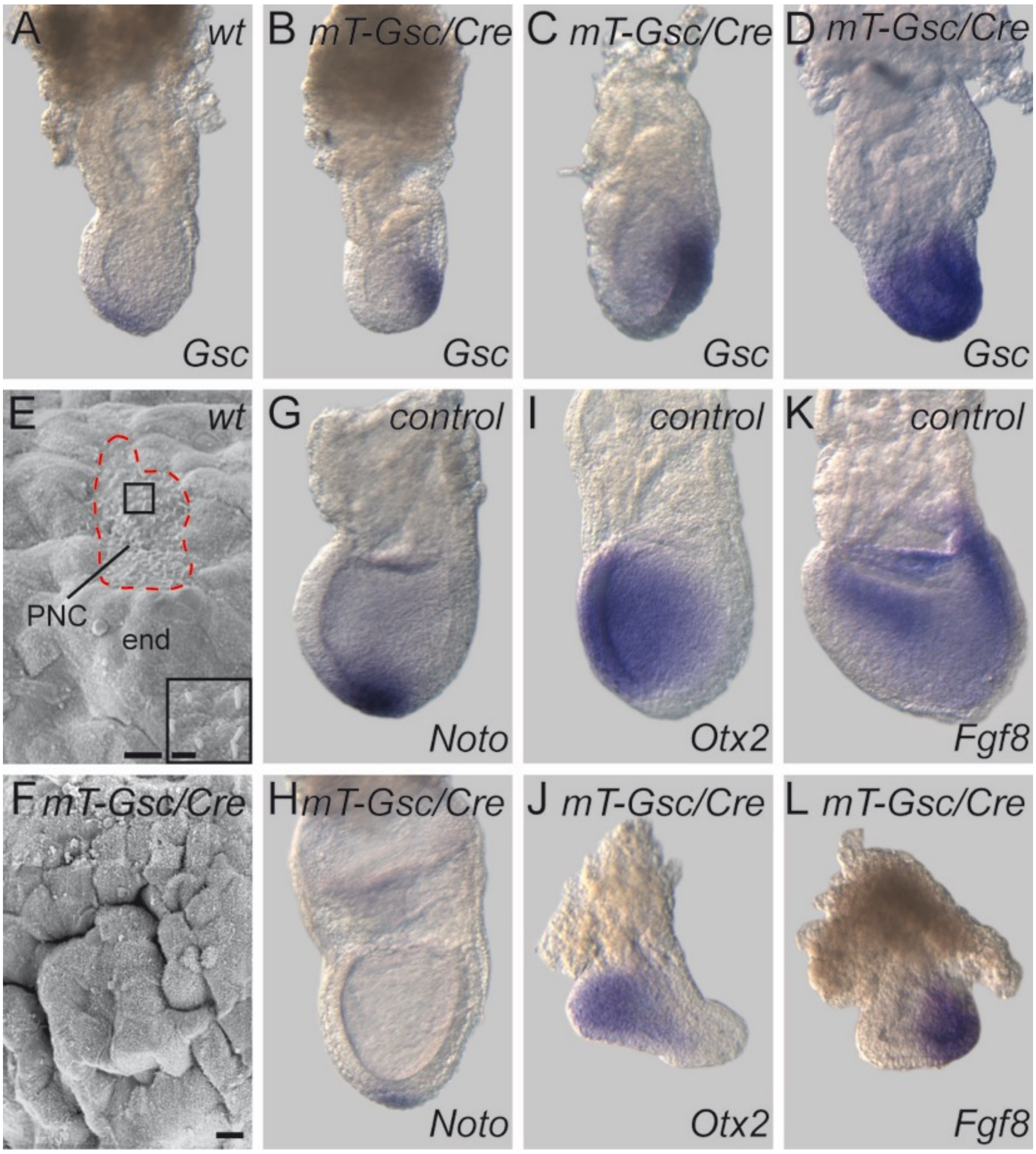

Fig. S3. Characterization of E7.5 *mt-Gsc/Cre* embryos.
(A-D) Variable degree of ectopic *Gsc* mRNA expression in E7.5 *mT-Gsc/Cre* embryos (B-D) compared to wildtype (A; wt).
(E, F) Scanning electron micrographs, revealing absence of ciliated epithelium of posterior notochord (PNC; outlined by dashed red line), and deep furrows in endodermal cell layer of mt-Gsc/Cre specimen (F) as compared to wt embryo (E). Detail of ciliated epithelium shown in higher magnification in inset of (E).
(G, H) Reduced *Noto* mRNA transcription in *mt-Gsc/Cre* (H) compared to wt (G) embryo.
(I-L) *Otx2* (I, J) and *Fgf8* (K, L) gene expression demonstrate normal anterior-posterior axis specification in *mT-Gsc/Cre* (J, L) compared to wt (I, K) embryos.
Scale bars in (E, F) represent 10 µm and 2 µm in inset of (E).

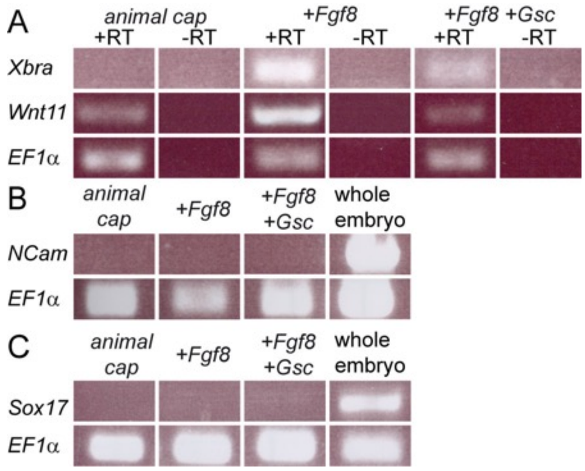

Fig. S4. Mesodermal differentiation of *Fgf8*-injected animal cap explants.
Semi-quantitative RT-PCR analysis of animal cap explants injected with *Gsc-GR* and/or *Fgf8*.
(A) *Fgf8* induces transcription of mesodermal marker genes *Xbra* und *Wnt11*.
(B, C) No induction of the neural marker *Ncam* (B) or the endoderm gene *Sox17* (C). Elongation factor 1α (*EF1α*) served as loading control.

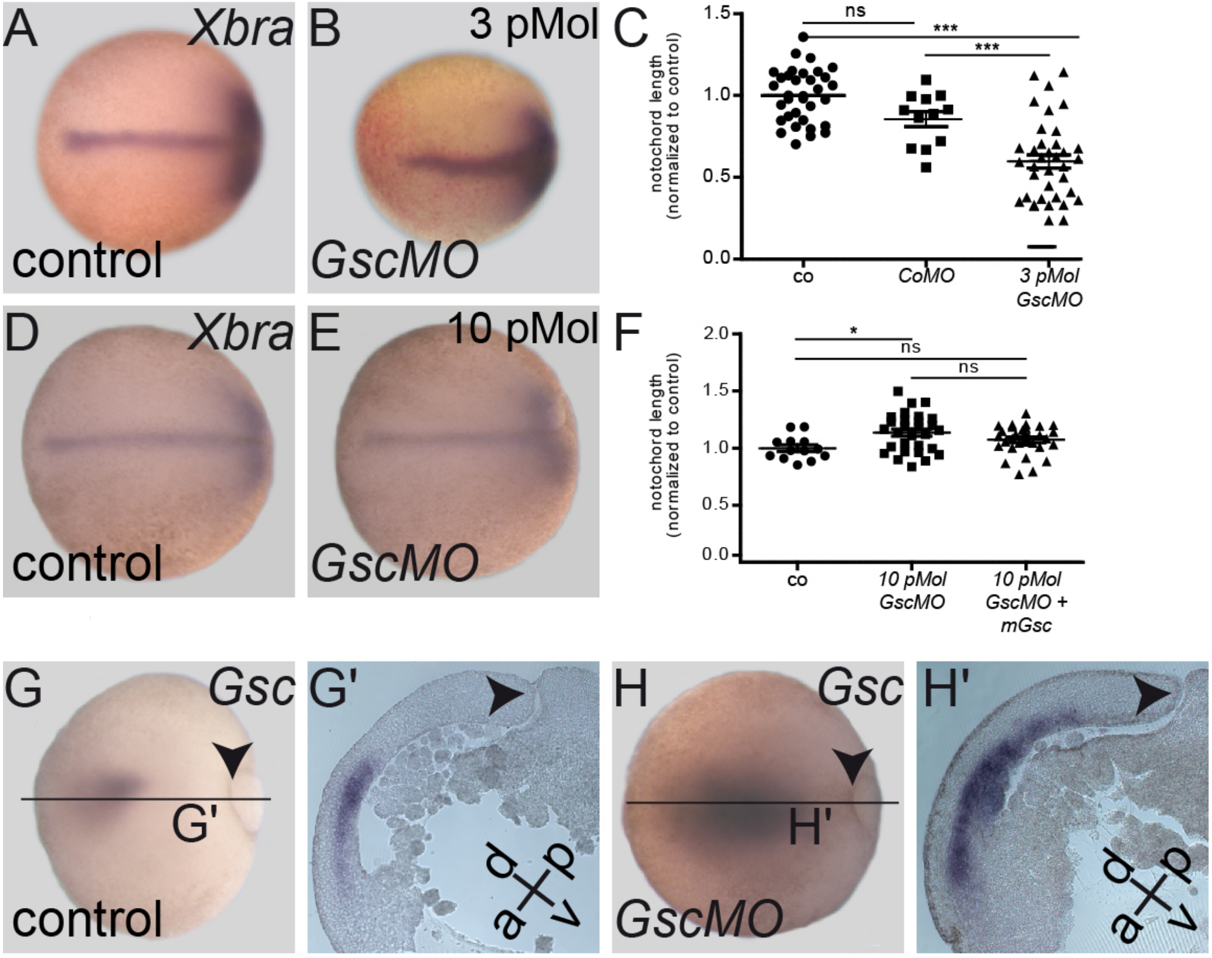

Fig. S5. CE phenotypes in *Gsc* morphant *Xenopus* embryos.
(A-C) Shorter and widened notochord as judged by *Xbra* mRNA expression in low dose *Gsc* morphant (B) as compared to control MO injected specimen (A). (C) Quantitative assessment of notochord lengths (normalized to control which was set to 1.0 in uninjected specimens).
(D-F) High dose injections of *GscMO* enhanced axis elongation. (D, E) *Xbra* mRNA expression in control un-injected embryo (co, D) and high dose *Gsc* morphant (E). (F) Quantification of notochord lengths in controls, high dose *Gsc* morphants and morphant specimens co-injected with a mouse *Gsc* cDNA construct.
(G, H) *Gsc* mRNA expression in low-dose *Gsc* morphants. (G, G‘) *Gsc* mRNA expression in control MO-injected neurula stage embryo. (H, H‘) Upregulated and expanded *Gsc* expression levels in *Gsc* morphant. Note that *Gsc* expression in morphant almost extended to the blastopore (arrowheads in G‘, H‘). a, anterior; co, control un-injected; CoMO; control MO-injected; d, dorsal; p, posterior; v, ventral. Embryos shown with anterior to the left.

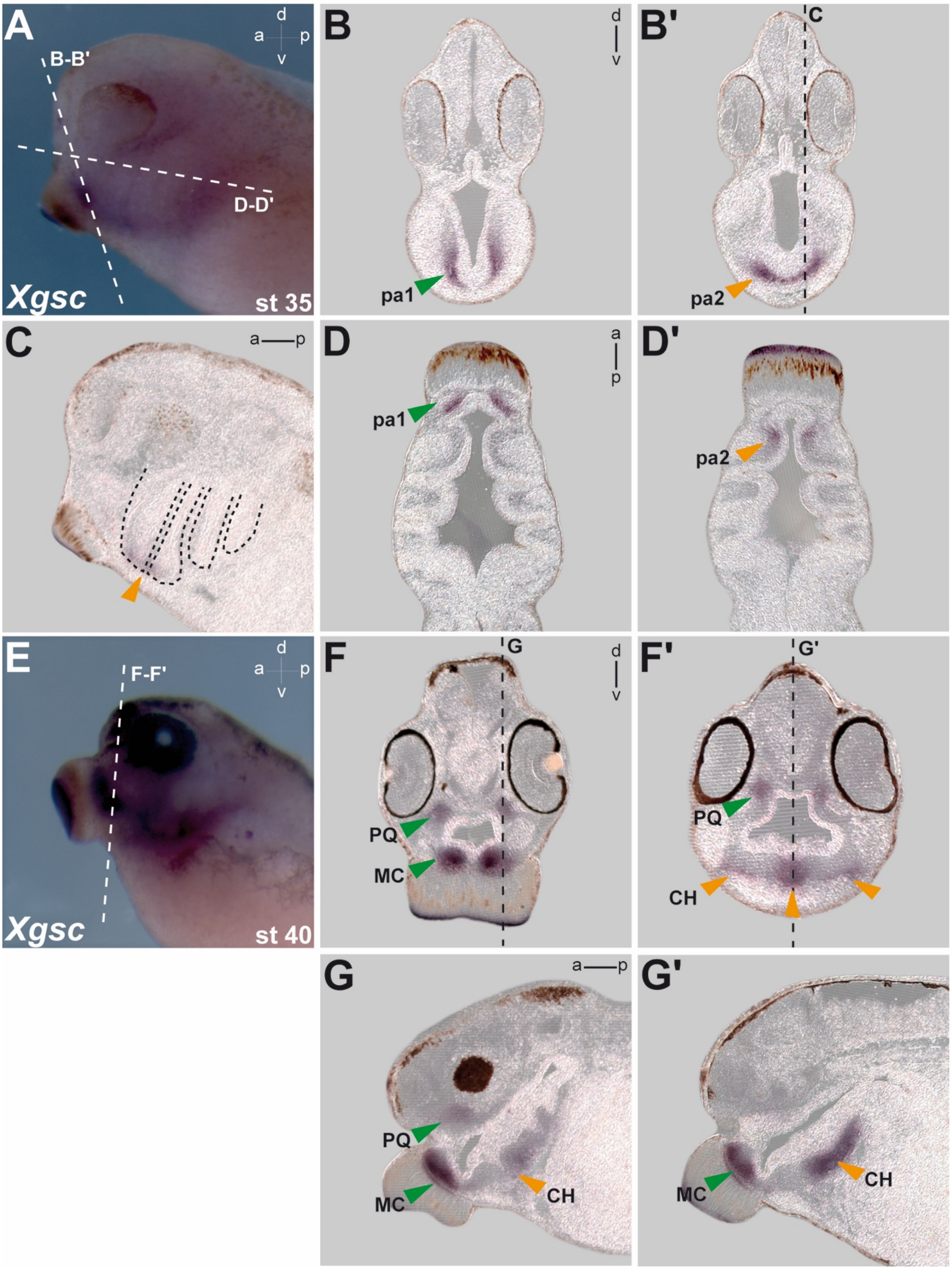

Fig. S6. *Gsc* expression in cranial neural crest and derived head cartilage. Whole-mount *in situ* hybridization of st. 35 (A-D) and st. 40 (E-G) tadpoles with a *Gsc*-specific antisense probe. Planes of histological vibratome sections are indicated by dashed lines. *Gsc* transcripts were found in pharyngeal arch (pa) mesenchyme and in differentiating cranial cartilage. Green arrowheads, pa1 and its derivatives palatoquadrate (PQ) and Meckel's cartilage (MC); orange arrowheads, pa2 and its derivative ceratohyale (CH).

```
Fruit fly:   MLAQMVETNSPPAGYTLKRSPSDLGEQQQPPRQISRSPGNTAAYHLTTAMLLNSQQCGYLGQRLQSVLQQQH

                                                   eh1/GEH
Human:       --------------------------------MPASMFSIDNILAARP-----------------------
Mouse:       --------------------------------MPASMFSIDNILAARP-----------------------
Chicken:     --------------------------------MPASMFSIDNILAARP-----------------------
Xenopus:     --------------------------------MPSGMFSIDNILAARP-----------------------
Zebrafish:   --------------------------------MPAGMFSIDSILAGRP-----------------------
Fruit fly:   AQHQQSQSQTPSSDDGSQSGVTILEEERRGGAAAASLFTIDSILGSRQQGGGTAPSQGSHISSNGNQNGLT
Tribolium:   AAPPRHHHQLLGNVFGE--GFKPVFKRGATPATATSLFTIDSILAPRPK----------------------
Platynereis: MYTDRMHHIPYYYQPVSAEQLKSG--ACPPAFSSP--FTIDSILAPRPL----------------------
Limpet:      TIAAMMPHLPYYYAAGQLKSCHSALMANSAAGGSPSLFSIDSILAPRP-----------------------

                                                                          X
Human:       RCKDSVLPVAHSAAAPVVFPALHGDSLYGASGGASSDYGAFYPRPVAPGGAGLPAAVSGSRLGYNNYFYGQL
Mouse:       RCKDAVLPVAPSAAAPVVFPALHGDSLYGAGGGTSSDYGAFYPRPVAPGGAGLPAAVGSSRLGYNSYFYGQL
Chicken:     RCKDSVLL---PPSAPVVFPSLHGDSLYGA----ASDYGGFYSRAVAPGSA-LPA-VGRSRLGYNNYYYGQL
Xenopus:     RCKESLLL---PQNGPLLFSSL-GESLYGP----A-DYSGFYNRTVAPTSA--LQGVNGSRLGYNNYYYGQL
Zebrafish:   SCKDSVLL---HRNAPVVFSNL-TESLYTA----AGDFNGLYSHTGPPAPN--LQSVNG-RIGYNNYYYGQL
Fruit fly:   SNGISLGLKRSGAESPASPNSNSSSSAAASPIRPQRVPAMLQHPGLHLGHLAAAAASGFAASPSDFL-VAYP
Tribolium:   ---------------------------PSSPQRP-----VLQHPGLHLGHIAAAG--GFGASSADFLAMAYP
Platynereis: ----------------------------TFPPRPP----YFHYPAAAAAAAAAAAL--HHHPHADLLASAYP
Limpet:      ----------------------------VPAHRPT---AYFPYPGITPGPYDLFAA----------AAAAYP

                                            X
Human:       HVQAAPVGPACCGAVPPLGAQQCSCVPTPPGYEGPGSVLVSPVPHQMLPYMNVGTLSRTELQLLNQLHCRR
Mouse:       HVQAAPVGPACCGAVPPLGAQQCSCVPTPPGYEGPGSVLVSPVPHQMLPYMNVGTLSRTELQLLNQLHCRR
Chicken:     HVATSPVGPSCCGAVPPLGAQQCSCVP-PAGYEGAGSVLMSPVPHQMLPYMNVGTLSRTELQLLNQLHCRR
Xenopus:     HLQT-PVGPSCCGAVQALGTQQCSCVPSATAYDGAGSVLIPPVPHQMLPYMNVGTLSRTELQLLNQLHCRR
Zebrafish:   HVQG-PTGPACCGAIPTLGSQQCPCIP--TGYDSAGSVLISPVPHQMMSYMNVGTLSRTELQLLNQLHCRR
Fruit fly:   NFYPNYMHAAAVAHVAAAQMQAHVSGAAAGLSGHGHHPHHPHGHPHHPHLGAHHHGQHHLSHLGHGPPPKR
Tribolium:   GLYPGYVHALAAASQAASMAGSHPQHQPPKRYPGITPGPYDLFAAAAAAYPSPFPGFLSPADLARAGQ-KR
Platynereis: GAFPGLMAADLMRV---------------------------------------------------GQKR
Limpet:      SPFPGFLSPADLARA--------------------------------------------------GQKR

                                         HD                                  Y
Human:       KRRHRTIFTDEQLEALENLFQETKYPDVGTREQLARKVHLREEKVEVWFKNRRAKWRRQKRSSSEESENAE
Mouse:       KRRHRTIFTDEQLEALENLFQETKYPDVGTREQLARKVHLREEKVEVWFKNRRAKWRRQKRSSSEESENAE
Chicken:     KRRHRTIFTDEQLEALENLFQETKYPDVGTREQLARKVHLREEKVEVWFKNRRAKWRRQKRSSSEESENAQ
Xenopus:     KRRHRTIFTDEQLEALENLFQETKYPDVGTREQLARKVHLREEKVEVWFKNRRAKWRRQKRSSSEESENAQ
Zebrafish:   KRRHRTIFTDEQLEALENLFQETKYPDVGTREQLARRVHLREEKVEVWFKNRRAKWRRQKRSSSEESENSQ
Fruit fly:   KRRHRTIFTEEQLEQLEATFDKTHYPDVVLREQLALKVDLKEERVEVWFKNRRAKWRKQKREEQERLRKLQ
Tribolium:   KRRHRTIFSEEQLEQLEATFEQTHYPDVVLREQLALKVDLKEERVEVWFKNRRAKWRKQKREEQERMRKIQ
Platynereis: KRRHRTIFTEEQLEALEATFAKTHYPDVMLREELAMKVDLKEERVEVWFKNRRAKWRKQKREEEARRRAAG
Limpet:      KRRHRTIFTEEQLEPLESTFHETHYPDVLLREELAIKVDLKEERVEVWFKNRRAKWRKTKREEEAARRAQK

                         Y
Human:       KWNKTSSSKASPEKREEEGKSDLDSDS
Mouse:       KWNKTSS-KASPEKREEEGKSDLDSDS
Chicken:     KWNKAS--KTSPEKRQEDGKSDLDSDS
Xenopus:     KWNKSS--KNSAEKRDEQAKSDLDSDS
Zebrafish:   KWNKST--KTTSEKIEE-GKSDVDSDS
Fruit fly:   EEQCGSTTNGTTNSSSGTTSSTGNGSLTVKCPGSDHYSAQLVHIKSDANGYSDADESSDLEVA
Tribolium:   EEDVCR---------------------------------SVEQQRLLQPQN-FSDED-SSDLEVA
Platynereis: TSDGASSSTVSNSPETSSDKSPHDDKSNSDVEEEDRLSHVDGEEISVTDDEDDVTGRNSLLSR
Limpet:      GLEPEKNITESDADVSICVDDETKDNNDCDSHESVNVDSCDELSQTAKSDDDDL---------

Platynereis: EYMTSHSESSASYRNELCSRPLSDNFSKSNFNTSSQSSCSSPSP
Limpet:      ----SDNDFSAS-----------------SPHSKQLDSPGPSSTS
```

Fig. S7. Multiple sequence alignment of Gsc proteins. Note that the eH1- and homeodomains are very highly conserved throughout the animal kingdom. Two similarly highly conserved domains N- and C-terminal of the homeodomain, marked "X" and "Y", are specific for vertebrate Gsc protein sequences. The following sequences were used: human (NM_173849.2), mouse (NM_010351.1), chicken (NP_990662.1), *Xenopus* (XM_018231890.1), zebrafish (NM_131017.1), fruit fly (CAA64699.1), tribolium (XP_008198241.1), *Platynereis* (AJ289023.1), limpet (AJ507423.1). Alignments were generated using multalin (http://multalin.toulouse.inra.fr/multalin/).

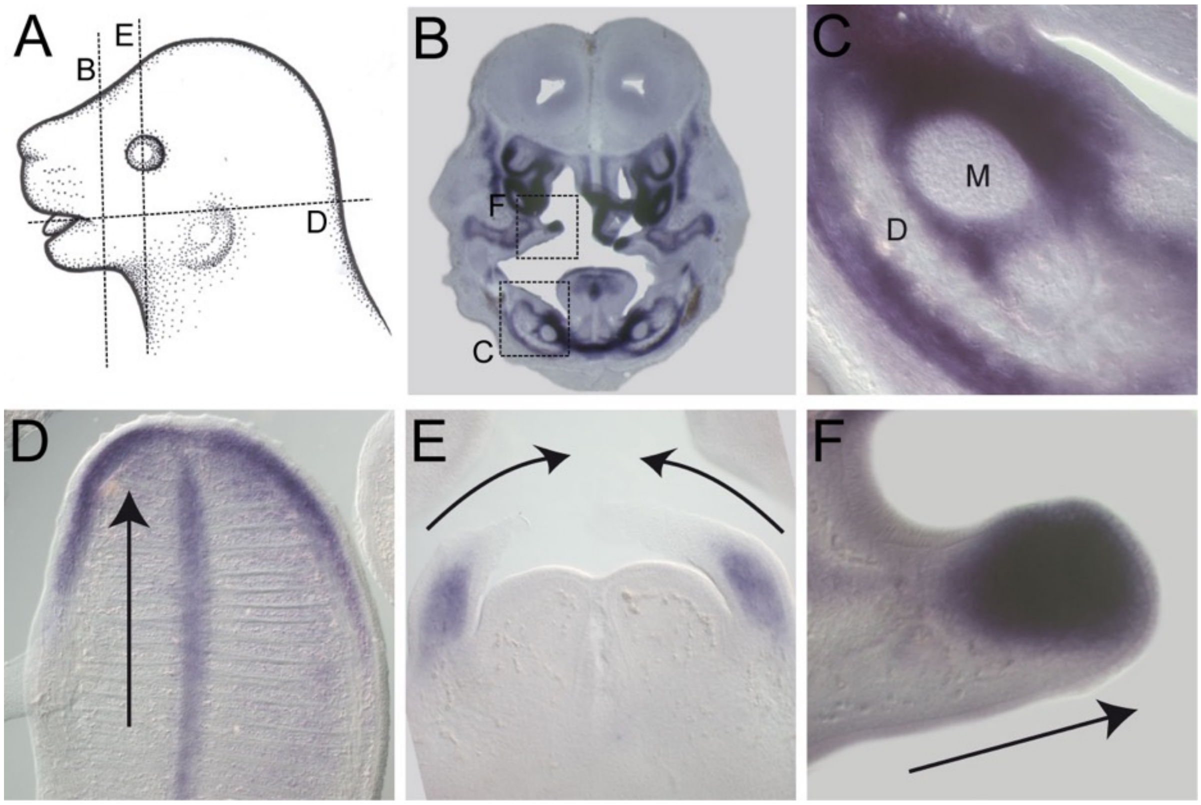

Fig. S8. *Gsc* expression adjacent to tissues undergoing elongation in E14.5 mouse embryos.
In situ hybridisation using a mouse *Gsc* antisense RNA probe on frontal and transversal vibratome section of E14.5 wildtype mouse embryos.
(A) Schematic depiction of sectional planes.
(B, C) *Gsc* transcripts localize to regions around Meckel's (M) cartilage and the developing *dentale*.
(D) *Gsc* was expressed at the tip of the tongue and the developing *septum linguae*. (E) *Gsc* transcript localization to the arytenoid swellings before fusion.
(F) *Gsc* expression in the mesenchyme of the palatal shelves.

	wt		BPD		NTD		n	n defects	p wt~defects
***GscGR*, 10 experiments**	n	%	n	%	n	%			Chi-Square
uninjected controls with/without dex	267	93,0	3	1,0	17	5,9	287	20	
GscGR without dex	183	92,0	6	3,0	10	5,0	199	16	
GscGR + dex st 6-8	67	38,3	64	36,6	44	25,1	175	108	<10-4
GscGR + dex st 11	32	47,8	7	10,4	28	41,8	67	35	<10-4
GscGR + dex st 12,5	46	97,9	1	2,1		0,0	47	1	0,080
***RhoAca*, 4 experiments**									
uninjected controls with/without dex	95	95,0	5	5,0	0	0,0	100	5	
GscGR without dex	20	80,0	1	4,0	4	16,0	25	5	
GscGR and *RhoAca* without dex	51	73,9	4	5,8	14	20,3	69	18	
GscGr with dex	20	22,2	61	67,8	9	10,0	90	70	
Rescue (*GscGR* and *RhoAca* with dex)	60	48,0	24	19,2	41	32,8	125	65	<10-4
***RhoAdn*, 4 experiments**									
uninjected controls with dex	149	96,1	3	1,9	3	1,9	155	6	
GscGR 160 pg without dex	85	88,5	4	4,2	7	7,3	96	11	
GscGr 160 pg with dex	65	58,6	20	18,0	26	23,4	111	46	
GscGR and *RhoAdn* without dex	55	47,4	37	31,9	24	20,7	116	61	
Rescue (GscGR and RhoAdn with dex)	22	21,8	61	60,4	18	17,8	101	79	<10-4
***Prickle*, 4 experiments**									
uninjected controls without dex	137	95,8	2	1,4	4	2,8	143	6	
GscGR without dex	70	93,3	5	6,7	0	0,0	75	5	
GscGR and *Prickle* without dex	70	82,4	9	10,6	6	7,1	85	15	
GscGr with dex	18	22,0	49	59,8	15	18,3	82	64	
Rescue (GscGR and Prickle with dex)	43	48,9	35	39,8	10	11,4	88	45	<10-4
***Vangl2*, 6 experiments**									
uninjected controls without dex	115	95,8	0	0,0	5	4,2	120	5	
GscGR without dex	55	94,8	2	3,4	1	1,7	58	3	
GscGR and *Vangl2* without dex	90	72,0	10	8,0	25	20,0	125	35	
GscGr with dex	38	37,3	48	47,1	16	15,7	102	64	
Rescue (GscGR and Vangl2 with dex)	70	55,6	40	31,7	16	12,7	126	56	0,006
***T*, 6 experiments**									
uninjected controls without dex	131	94,9	2	1,4	5	3,6	138	7	
GscGR without dex	37	90,2	2	4,9	2	4,9	41	4	
GscGR and *T* without dex	82	79,6	8	7,8	13	12,6	103	21	
GscGr with dex	27	20,9	82	63,6	20	15,5	129	102	
Rescue (*GscGR* and *T* with dex)	53	35,3	44	29,3	53	35,3	150	97	0,008
***Wnt11*, 3 experiments**									
uninjected controls	45	90,0	1	2,0	4	8,0	50	5	
GscGR without dex	20	80,0	4	16,0	1	4,0	25	5	
GscGR and *Wnt11* without dex	23	69,7	3	9,1	7	21,2	33	10	
GscGR with dex	8	11,0	54	74,0	11	15,1	73	65	
Rescue (*GscGR* and *Wnt11* with dex)	31	33,3	38	40,9	24	25,8	93	62	0,007

Table S1. *Gsc-GR* induced CE phenotypes and rescue by PCP components.
Raw data of experiments summarized in Figure 2. BPD, blastopore closure defect; NTD, neural tube closure defect. Statistical analyses were performed using StatPages.org.
